# Bridging the gap between gene expression and metabolic phenotype via kinetic models

**DOI:** 10.1186/1752-0509-7-63

**Published:** 2013-07-22

**Authors:** Francisco G Vital-Lopez, Anders Wallqvist, Jaques Reifman

**Affiliations:** 1 DoD Biotechnology High Performance Computing Software Applications Institute, Telemedicine and Advance Technology Research Center, U.S. Army Medical Research and Materiel Command, Ft. Detrick, MD, 21702, USA

**Keywords:** Gene expression, Kinetic models, Metabolic networks, *S. cerevisiae*, Transcriptomics, Fluxomics, Metabolomics

## Abstract

**Background:**

Despite the close association between gene expression and metabolism, experimental evidence shows that gene expression levels alone cannot predict metabolic phenotypes, indicating a knowledge gap in our understanding of how these processes are connected. Here, we present a method that integrates transcriptome, fluxome, and metabolome data using kinetic models to create a mechanistic link between gene expression and metabolism.

**Results:**

We developed a modeling framework to construct kinetic models that connect the transcriptional and metabolic responses of a cell to exogenous perturbations. The framework allowed us to avoid extensive experimental characterization, literature mining, and optimization problems by estimating most model parameters directly from fluxome and transcriptome data. We applied the framework to investigate how gene expression changes led to observed phenotypic alterations of *Saccharomyces cerevisiae* treated with weak organic acids (i.e., acetate, benzoate, propionate, or sorbate) and the histidine synthesis inhibitor 3-aminotriazole under steady-state conditions. We found that the transcriptional response led to alterations in yeast metabolism that mimicked measured metabolic fluxes and concentration changes. Further analyses generated mechanistic insights of how *S*. *cerevisiae* responds to these stresses. In particular, these results suggest that *S*. *cerevisiae* uses different regulation strategies for responding to these insults: regulation of two reactions accounted for most of the tolerance to the four weak organic acids, whereas the response to 3-aminotriazole was distributed among multiple reactions. Moreover, we observed that the magnitude of the gene expression changes was not directly correlated with their effect on the ability of *S*. *cerevisiae* to grow under these treatments. In addition, we identified another potential mechanism of action of 3-aminotriazole associated with the depletion of tetrahydrofolate.

**Conclusions:**

Our simulation results show that the modeling framework provided an accurate mechanistic link between gene expression and cellular metabolism. The proposed method allowed us to integrate transcriptome, fluxome, and metabolome data to determine and interpret important features of the physiological response of yeast to stresses. Importantly, given its flexibility and robustness, our approach can be applied to investigate the transcriptional-metabolic response in other cellular systems of medical and industrial relevance.

## Background

It is well known that cells regulate gene expression to carry out different functions depending on their physiological state and environment. However, it is less well understood how this regulation is orchestrated and how gene expression changes drive cells to adapt particular phenotypes. Developments in high-throughput technologies have contributed to answer these questions by generating a wealth of data on different cellular components and processes (e.g., transcriptome, proteome, metabolome, fluxome, and protein-protein interaction data). Hence, one of the challenges in systems biology is how to integrate and analyze such data to elucidate the underlying cellular physiology. In particular, the development of genome-scale computational models and analysis tools can help expand our understanding of how gene transcription alters cellular metabolism.

Different approaches have already made considerable headway in integrating gene expression and metabolism [1-6]. Perhaps the most developed efforts are based on combining stoichiometric models of metabolic networks and gene expression data. In these approaches, gene expression levels are used to parameterize the flux capacity of metabolic reactions to create context-specific models [7-9]. For example, we followed this approach to characterize the metabolic adaptations of *Mycobacterium tuberculosis* to hypoxia and identify metabolic alterations required for adaptation to a lifestyle of low metabolic activity [10]. Alternatively, computational approaches have been developed to infer regulatory networks from gene expression data [11], which in turn have been integrated with metabolic network models to describe the adaptation of an organism to different conditions [12-15].

Combining stoichiometric models of metabolic networks and gene expression data has proven useful in analyzing transcriptome, proteome, and fluxome data but presents limitations in analyzing metabolome data. These limitations can be overcome using kinetic models, in which metabolite concentrations are the primary variables as opposed to fluxes in constraint-based methods. However, the use of large-scale kinetic models (i.e., with hundreds of reactions) has been daunted by the general belief that the chances of obtaining a useful model, given the lack of accurate reaction rate expressions and kinetic parameters, are low. This paradigm has begun to change due, in part, to the high-throughput techniques that have increased the abundance, quality, and scope of the data needed for model construction.

In addition to data availability, there are two other factors, arising from the biology of the systems, that ease the construction of large-scale kinetic models [16]. The first one is the observation that the structure of a biological network (i.e., what the network components are and how they are connected) largely determines its function, as observed in constraint-based analyses [17]. Thus, the available reconstructions of metabolic networks provide us with more than a solid scaffold to construct kinetic models: the performance of the network is confined within well-characterized limits. The second factor is the “sloppiness” of parameter sensitivities, which seems to be a widespread property of models of biological systems [18]. This sloppiness property implies that most of the model parameters cannot be collectively estimated with certainty, even by fitting large amounts of “ideal” data. Paradoxically, it also implies that knowledge of the precise value of most parameters is not critical for describing a system’s behavior. Motivated by these factors, methods to construct large-scale kinetic models of metabolism have started to emerge [19-22].

In this work, our objective was to investigate how the response of a cell to a perturbation (in terms of transcriptome or proteome data) induces changes in its phenotype (in terms of fluxome and metabolome data). For this purpose, we developed a computational approach based on kinetic models that provides a mechanistic link between transcriptional regulation and metabolism. Our proposed modeling framework overcomes the major obstacles in the construction of large-scale kinetic models of metabolism, namely, the detailed definition of appropriate reaction rate expressions and the determination of model parameters. As in previous approaches [19-22], we automatically translated a metabolic network model into a kinetic model using generic expressions, a particular case of generalized mass action (GMA) kinetics, for the reaction rates [23]. However, in contrast to these approaches, our method does not require extensive parameter estimation, mining the literature, or using random-sampling schemes to obtain parameter values. Most of the model parameters are obtained directly from experimental data that are routinely available (i.e., protein or gene expression data and flux distributions or uptake/production rates). Although the models could be used to investigate dynamic behavior, this would require additional input parameters in terms of an extensive set of metabolite concentrations. However, as these data are typically not available, and similar to other approaches such as ensemble modeling [20], we have used the proposed models to describe and analyze steady-state behavior.

Here, we constructed kinetic models to analyze the steady-state metabolism of *S*. *cerevisiae* based on two independent studies in which the transcriptional and metabolic responses to treatment were measured in chemostat cultures with weak organic acids (WOAs) [24] and under histidine starvation conditions [25]. The simulation results demonstrated that integration of gene expression with metabolic network models enabled us to capture aspects of the response of *S*. *cerevisiae* that would not have been possible by the independent analyses of the gene expression data or the metabolic network alone.

## Methods

### Model construction

**Figure 1:**
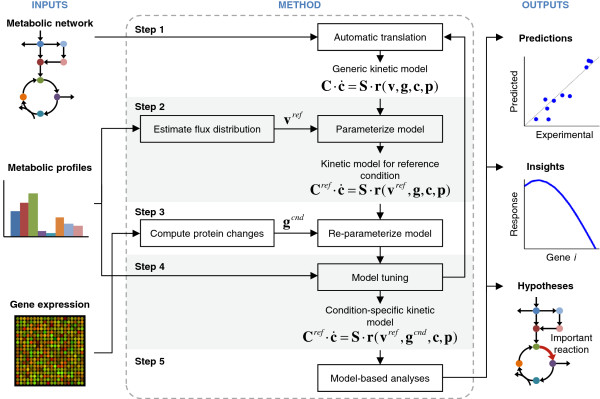
**Construction of large-scale kinetic models using commonly available information and data.** The method starts with the automatic translation of a metabolic network reconstruction into a generic kinetic model, which is parameterized using the metabolic profile for a reference condition (*ref*). The kinetic model is parameterized to simulate other conditions using gene expression profiles and tuned using the metabolic profiles for the conditions of interest (*cnd*). The tuned model can be used to perform different model-based analyses. **C** denotes a diagonal matrix with elements equal to the absolute metabolite concentrations, **c** represents the vector of normalized metabolite concentrations and **ċ** denotes its time derivative, **S** denotes the stoichiometric matrix of the metabolic network reconstruction, **r** represents the vector of reaction rates, **v** denotes the flux distribution, **g** represents the vector of gene expression ratios, and **p** represents a vector of other condition-specific model parameters.

Figure 1 shows the workflow of the proposed method for constructing large-scale kinetic models of metabolism. In this section, we present the basic details of the method; their rationale and derivation are provided in Additional file 1. The method requires three inputs: a metabolic network reconstruction, metabolic flux distribution at a reference condition, and gene expression profiles for the reference condition and other condition of interest. In Step 1, the metabolic network reconstruction was translated into a kinetic model using a particular case of GMA kinetics that allowed us to lump several parameters into a single parameter that can be estimated from metabolic flux measurements (see Additional file 1). These rate expressions also allowed us to parameterize the model to simulate other conditions using gene expression data. We used different expression forms for irreversible and reversible reactions. For irreversible reactions (given the general lack of knowledge of allosteric regulations), we assumed that products inhibit the reaction rate to allow reactions downstream of an irreversible reaction to have an effect on the flux through a pathway. Thus, for a general irreversible reaction:

(1) $$\sum_{i}a_{i}A_{i}\to \sum_{j}b_{j}B_{j},$$

we used the expression form:

(2) $$r=vg{\left(\frac{{\prod_{i\;}\left[A_{i}\right]}^{m_{i}}}{{\prod_{j}\left[B_{j}\right]}^{m_{j}}}\right)}^{1/Y},$$

where *a*_*i*_ and *b*_*j*_ denote the stoichiometric coefficients of species *A*_*i*_ and *B*_*j*_ in the reaction, respectively, *r* represents the reaction rate, the parameter *v* denotes the value of the reaction rate or flux through the reaction at a reference condition, *g* represents the overall gene expression ratio (between a condition of interest and a reference condition) of the genes associated with the reaction, and the square brackets denote normalized metabolite concentrations. The constants *m*_*i*_ (*m*_*j*_) are set to 2.0 if *a*_*i*_ (*b*_*j*_) is 2.0, or to 1.0 otherwise. This choice of the constants *m*_*i*_ (*m*_*j*_) is arbitrary, however, as shown in the Results Section, it has a minor effect on the simulation results. For lumped reactions (the combination of multiple sequential reactions into a single overall reaction), *γ* denotes the number of irreversible steps and *γ=*1 for individual reactions. A lumped reaction is irreversible if at least one of its steps is irreversible. Note that the actual reaction rates depend linearly on the protein levels. However, in this work, we have substituted protein levels with the more readily available gene expression data. This approximation is supported by experimental evidence that shows a moderate correlation between protein and mRNA expression ratios for *S. cerevisiae*[26,27]. As shown in the Results Section, the quality of our simulation results suggests that this approximation is acceptable for the experiments analyzed here.

Similarly, for a general reversible reaction, we used the expression form:

(3) $$r=g\left(v^{f}{\prod_{i}\left[A_{i}\right]}^{m_{i}}-v^{b}{\prod_{j}\left[B_{j}\right]}^{m_{j}}\right),$$

where *v*^*f*^ and *v*^*b*^ were determined as follows:

(4) $$v^{f}=\mathrm{\beta v}\;\mathrm{and}\;v^{b}=\left(\beta -1\right)v,\;\mathrm{if}\;v>0,$$

(5) $$v^{f}=\left(\beta -1\right)v\;\mathrm{and}\;v^{b}=\mathrm{\beta v},\;\mathrm{if}\;v<0,$$

where *β* is a parameter that relates the rate of the forward and backward reactions to the overall flux at the reference condition. The value of *β* depends on the equilibrium constant and on the reactants concentration at the reference condition. If these data are not available, as in the experiments analyzed here, *β* could be estimated by a fitting procedure using other available data. However, to avoid over fitting and assuming that the model behavior has low sensitivity to individual *β*s we started by approximating all *β*s with the same value (except for reactions in parallel routes that must satisfy additional thermodynamic constraints as detailed in Additional file 1) and checked whether it was necessary to estimate individual *β*s. As shown in the Results Section, there was no need for estimating individual *β*s as a single-value approximation proved satisfactory.

All reactions are described using these rate expression forms, except for the modeling of the biomass growth rate. A mechanistic representation that takes into account most of the factors influencing the growth rate is currently unfeasible. Therefore, we followed a heuristic approach to define a kinetic expression for the growth rate that is compatible with the observations from the analyzed experiments in this work [25]. Instead of defining a single reaction representing the formation of biomass, we included drain fluxes for each of the biomass precursors as in the model of Moxley et al. [25] and defined the biomass growth rate as follows:

(6) $$\mu =\lambda X_{\mathrm{MW}}\sum_{i}\phi_{i}r_{i},$$

where *μ* denotes the growth rate, *λ* denotes a correction factor, *X*_*MW*_ denotes the biomass molecular weight, *ϕ*_*i*_ denotes the moles of carbon per mole of biomass precursor, and the summation included only the drain fluxes to biomass. Note that this definition of the biomass growth rate can predict a non-zero rate even if some of the drain fluxes are zero. However, such extreme cases were not observed in the simulations carried out in this paper.

The drain fluxes to biomass included overall reactions for one-carbon metabolism, synthesis of lipids, carbohydrates, and RNA, as well as individual reactions for each amino acid. The reaction rate for the drain fluxes of each amino acid was defined as follows:

(7) $$r=\mathrm{vg}\left(\prod_{i}\left[A_{i}\right]\right){\;}^{\alpha }\min_{j}{\left(\left[A_{j}\right]\right)}^{\alpha },$$

where the value of *α* was chosen such that the drain fluxes have low sensitivity to changes in precursor concentrations, based on the measurements from Moxley et al. [25]. The subscript *i* comprises the metabolites consumed in the drain flux (i.e., one amino acid and ATP) and the subscript *j* comprises all the amino acids. The second factor, representing the lowest concentration of any amino acid, was included based on the assumption that a low concentration of any amino acid would slow down protein synthesis and therefore the drain fluxes of the other amino acids. The reaction rate for the other drain fluxes was defined as follows:

(8) $$r=\mathrm{vg}\left(\prod_{i\;}\left[A_{i}\right]\right){\;}^{\alpha },$$

where the subscript *i* comprises the metabolites consumed in each drain flux.

The end product of Step 1 is a kinetic model describing the mass balances of the metabolites in the metabolic network and it is derived directly from the network reconstruction, which provides the stoichiometry of each reaction, and the rate expressions obtained from Eqs. 2, 3, 6, 7, and 8. The kinetic model can be represented as:

(9) $$C\cdot \dot{c}=S\cdot r\left(v,g,c,p\right),$$

where **C** is a diagonal matrix with elements equal to the absolute metabolite concentrations used for normalization, **c** represents the vector of normalized metabolite concentrations and **ċ** denotes its time derivative, **S** denotes the stoichiometric matrix of the metabolic network reconstruction, **r** represents the vector of reaction rates, **v** denotes the flux distribution, **g** represents the vector of gene expression ratios, and **p** denotes a vector of the other model parameters (i.e., *α*, *β*, *λ*, and other condition-specific parameters). Under steady-state conditions, **C** is not required and, thus, for steady-state analysis, the only parameters to be estimated are **v**, **g**, and **p.**

In Step 2, we parameterized the model for the reference condition. Using the reference condition for normalizing the metabolite concentrations and gene expression levels, both **c** and **g** become equal to 1.0, and **r**=**v**^*ref*^, where **v**^*ref*^ is the flux distribution at the reference condition. Therefore, for steady-state analysis, the model for the reference condition was parameterized with **v**^*ref*^. A flux distribution determined using ^13^C-labeling experiments provides a good estimate of **v**^*ref*^. If such flux distribution is not available, a reasonable estimate can be obtained using exchange fluxes, as described in Additional file 1.

Another notable feature of the method is that the model can be parameterized to simulate other conditions using the gene expression ratio between the condition of interest and the reference condition (Step 3). We assumed that relative changes in gene expression led to similar relative changes in protein abundance and we neglected post-translational and other regulatory mechanisms of enzymatic activity. Note that, if available, proteome data can be used instead of gene expression data. For reactions associated with multiple genes, we computed an overall gene expression change as described in Additional file 1.

In Step 4, we tuned the constructed models by comparing model predictions (i.e., metabolic fluxes and metabolite concentration changes) with experimental measurements. We resolved detected inconsistencies until the model gave satisfactory performance. Finally, once its performance is satisfactory, the model can be used to carry out different model-based analyses, such as predicting non-measured variables, determining the effect of a particular expression level on a given metabolic function, or to identify important reactions in the network (Step 5).

### Experimental data

The experimental data for the analysis of the response of *S. cerevisiae* to treatment with a WOA (acetic acid, benzoic acid, propionic acid, or sorbic acid) were obtained from Abbott et al. [24]. To compute the gene expression ratios from the raw intensity values, the microarray data were scaled such that the average intensity for each microarray was 150.0. For each condition, the median intensity (of three replicates) was used as the expression level of every gene in each condition. Every treatment-reference condition pair was smoothed using the Lowess smoothing method [28] and the gene expression ratios were computed from the smoothed data. The uptake rate of glucose and production rates of ethanol, glycerol, lactic acid, acetic acid, and CO_2_ were reported, as well as the biomass yield and concentration, and the extracellular glucose concentration for each treatment condition.

For the analysis of the metabolic response induced by the Gcn4 regulator, the experimental data were taken from Moxley et al. [25]. They report the gene expression data, flux distribution estimated from ^13^C-labeling experiments, and the concentration changes of 17 free amino acids for chemostat culture of wild-type and *gcn4*-knockout mutant strains of *S. cerevisiae*.

### Implementation and availability

Model construction, data processing, and simulations were carried out in MATLAB (2011b, The MathWorks Inc., Natick, MA). The kinetic model (MATLAB scripts and in SBML format) and parameter sets for simulating both experiments are provided in Additional file 2.

## Results

### Construction of large-scale kinetic models

**Figure 2:**
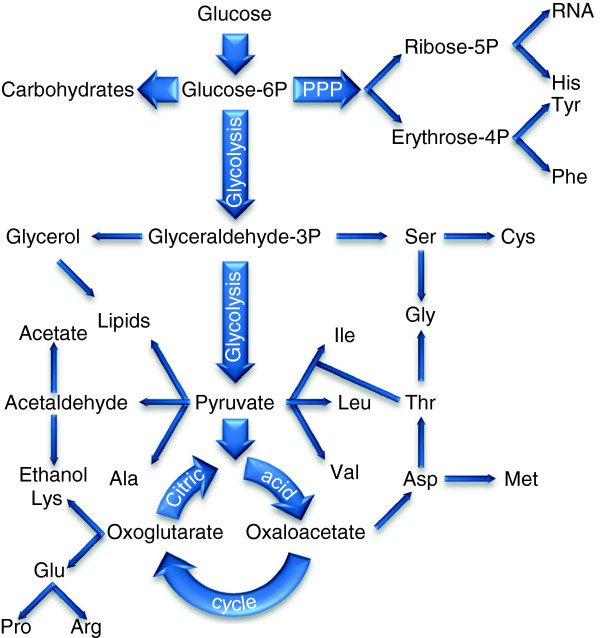
**Metabolic network of the central carbon metabolism and amino acids synthesis pathways of *S. cerevisiae*.** The network includes glycolysis, the pentose phosphate pathway (PPP), the citric acid cycle, and pathways for the synthesis of biomass precursors (i.e., amino acids, carbohydrates, lipids, and RNA). The figure shows a simplified diagram of the network. The actual network has 75 metabolites and 125 reactions.

We applied the method to construct condition-specific kinetic models of the metabolic network of *S. cerevisiae*. We constructed the metabolic network based on the network presented by Moxley et al. [25]. Figure 2 depicts the metabolic network, which includes the glycolysis pathway, the pentose phosphate pathway, the citric acid cycle, and pathways for the synthesis of biomass precursors (i.e., amino acids, carbohydrates, lipids, and RNA) and it has 75 metabolites and 125 reactions associated with 309 genes. We obtained the parameters **v** and **g** directly from experimental data [24,25] and the parameters in **p** were estimated as described in Additional file 1 and given in Table 1. Metabolite concentration changes **c** were computed by solving the model (Eq. 9) assuming steady state conditions in all simulations. We used the constructed models to analyze the transcriptional and metabolic responses of *S. cerevisiae* under histidine starvation conditions and to treatment with WOAs. The details of the metabolic network are given in Additional file 3.

**Table 1:** **Fitting parameters for the kinetic model of *S. cerevisiae *metabolic network**

**Parameter**	**Comment**	**Value**^**a**^	
		**3-AT**^b^	**WOA**^c^
*α*	Kinetic order for biomass drain fluxes	1.00×10^-1^	1.00×10^-1^
*β*^d^	Ratio of the forward (or backward if reference flux is negative) reaction rate	3.00×10^1^	3.00×10^1^
	to the overall rate of the reversible reactions		
*γ*	Correction factor for biomass growth rate	9.80×10^-1^	1.00×10^0^
*k*_*ser*→*gly*_	Inhibition factor of the synthesis of glycine from serine by 3-AT	2.27×10^-2^	-
*k*_*his*_	Inhibition factor of the synthesis of histidine 3-AT	4.87×10^-1^	-
*r*_*WOA*_	WOA uptake rate	-	3.00×10^1^

^a^ All parameters are dimensionless.

^b^ Parameter values for simulating the response of *S. cerevisiae* to 3-aminotriazole (3-AT).

^c^ Parameter values for simulating the response of *S. cerevisiae* to weak organic acids (WOA).

^d^ Reversibility of the reactions was based on the designations in the model by Moxley et al. [25] and the KEGG database (http://www.genome.jp/kegg/).

### Response of *S. cerevisiae* to histidine starvation

The activator protein Gcn4 of *S. cerevisiae* regulates the expression of nearly all genes encoding enzymes involved in amino acid synthesis under starvation conditions [29]. Moxley et al. [25] studied the regulatory and metabolic changes induced by Gcn4 under histidine-deficient conditions. Specifically, they cultivated wild-type and *gcn4*-knockout mutant (Δ*gcn4*) strains of *S. cerevisiae* in aerobic chemostats treated with 3-aminotriazole (3-AT), an inhibitor of imidazoleglycerol-phosphate dehydratase, the sixth step of the histidine synthesis pathway. The concentration of 3-AT was adjusted such that the Δ*gcn4* and wild-type cultures produced similar biomass levels and uptake and production rates of extracellular metabolites. They measured gene expression levels (See Additional file 1 for details about gene expression data processing), metabolic fluxes (using ^13^C-labeling experiments), and the intracellular concentration of free amino acids for each culture. We used the measured metabolic fluxes of the Δ*gcn4* culture as the reference condition **v**^*ref*^ and the gene expression ratios between the two cultures **g** to parameterize the model for simulating the wild-type cultures. The parameters in **p** used in these simulations are given in Table 1. First, we showed that the model was able to reproduce the experimental data. We then examined possible mechanisms of action of 3-AT and delineated the effect of the gene expression changes on yeast’s ability to grow when exposed to this drug.

#### The model quantitatively links gene expression regulation with metabolism

**Figure 3:**
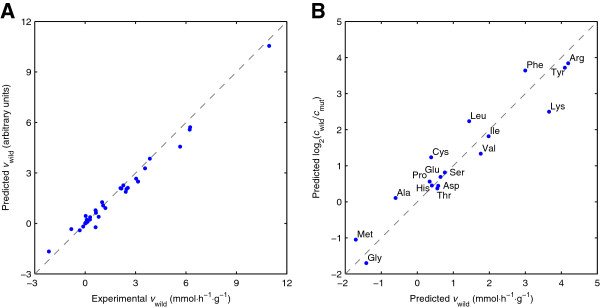
**Predicted metabolic response of the wild-type culture treated with 3-aminotriazole. (A)** Predicted metabolic fluxes plotted against experimental values. **(B)** Predicted concentration of free amino acids plotted against experimental values. The experimental data were taken from Moxley et al. [25].

Figure 3 shows the predicted metabolic fluxes and free amino acid concentrations for the wild-type culture. The Pearson’s correlation coefficient *ρ* between the predicted and measured metabolic fluxes was 0.99 and the slope of the best linear fit was 0.91. This high accuracy was due to the similarity between the experimental flux distributions of the reference (i.e., Δ*gcn4*) and the wild-type conditions (see Additional file 1). What we did not expect was the high accuracy of the predicted concentration changes. The *ρ* between the logarithmic ratios of the predicted and experimental concentration of free amino acids was 0.96, whereas the slope of the best linear fit was 0.86. This is noteworthy because the kinetic model (75 metabolites, 125 reactions) has only five fitting parameters (Table 1). Moreover, these simulation results were relatively insensitive to the particular choice of the constants *m*_*i*_ and *m*_*j*_ we used (Additional file 1).

Note that although we used the experimental concentration changes to estimate the value of the fitting parameters, the high *ρ* of 0.96 could not be achieved without parameterizing the model with the gene expression data. Nonetheless, the *ρ* without using the gene expression data was relatively high (*ρ* = 0.80; see Additional file 1). Based on these results, we can derive two general observations. First, the structure of the metabolic network, which we exploited to constrain the kinetic parameters **v** in our modeling framework, considerably contributed to the explanation of the experimental observations as we initially assumed. Second, gene expression changes were required to further improve the simulation results. Thus, both the metabolic network and the gene expression changes were required by the framework to establish the mechanistic link between gene expression regulation and metabolism.

#### Model-based identification of mechanisms of action of 3-AT

The proposed modeling framework can be used to investigate how a chemical agent acts on metabolism. The basic idea is that inconsistencies between model simulations and the experimental data could point out modeling errors or omissions that may be related to the mechanisms of action of the chemical agent. We proved this idea by showing that we were able to identify the known target of 3-AT. For this, we ranked the reactions according to how much their perturbations were able to reduce inconsistencies, i.e., the sum of squared errors (SSE) for the amino acid concentration changes. Briefly, we simulated the perturbation of each intracellular reaction (one reaction at a time and considering forward and backward rates individually) by multiplying its rate by a constant between 0.1 and 10.0 that minimized the SSE. Table 2 shows the top ten reactions whose perturbations resulted in the largest reductions of inconsistencies (the complete list is provided in Additional file 3). The known target of 3-AT was ranked third and other reactions of histidine synthesis and histidine flux toward biomass were ranked fourth and fifth, respectively. The first and second reactions were the forward and backward reactions of the glycine synthesis from serine. To further investigate these results, we independently predicted other enzymes besides the primary target that could be inhibited by 3-AT using in-house drug target identification approach [30]. We identified six plausible additional targets, five of which are associated with three different steps (including the limiting step) of the guanosine triphosphate synthesis pathway, which is a precursor for tetrahydrofolate synthesis. The other off-target candidate was dihydrofolatereductase, which catalyzes the last step of tetrahydrofolate synthesis (see Additional file 1). Tetrahydrofolate (which was not included in the model) is a coenzyme required for the synthesis of glycine from serine. This result reinforces the hypothesis that 3-AT may also be inhibiting the synthesis of glycine from serine at the high 3-AT concentration of the wild-type cultures. For simplicity, we assumed that the level of tetrahydrofolate is lower in the wild-type culture and modeled this hypothesis with a single parameter (*k*_*ser*→*gly*_, Table 1). These results highlight the potential of our method to predict mechanism of action of chemical agents by contrasting model simulations with experimental data.

**Table 2:** **Predicted targets of 3-aminotriazole in the *S. cerevisiae *central carbon metabolic network**

**Reaction index**^**a**^	**Reaction**	**Normalized SSE**^**b**^
46	SER ↔ GLY + METTHF (forward)	0.50
46	SER ↔ GLY + METTHF (backward)	0.53
79	R5P + METTHF + 2 ATP → IAP	0.56
91	HIS + ATP → HISBIO	0.60
80	IAP + GLU → HIS + AKG + 2 NADH	0.71
6	G3P ↔ PEP + NADH + ATP (forward)	0.71
6	G3P ↔ PEP + NADH + ATP (backward)	0.71
41	NADH → ATP	0.72
53	HSER + METTHF + ACCOA + 2 ATP → MET + ACE + 3 NADH	0.72
95	MET + ATP → METBIO	0.72

^a^ See Additional file 3 for details about the reactions and abbreviations.

^b^ The sum of squared errors (SSE) was normalized with the SSE of a simulation without perturbing any reaction.

#### Effect of gene expression regulation on the tolerance to 3-AT treatment

**Figure 4:**
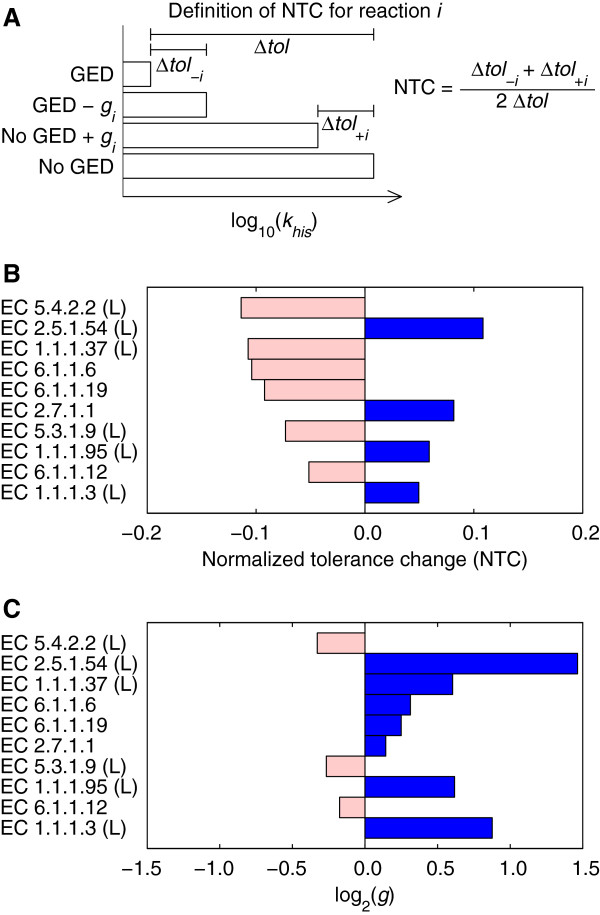
**Contribution of gene expression changes of individual reactions to 3-aminotriazole (3-AT) treatment tolerance. (A)** Definition of the metric used to compare the effects of gene expression changes of individual reactions. The normalized tolerance change (NTC) for reaction *i* was defined as the average of the changes (*Δtol*_*+i*_ and *Δtol*_*-i*_) in the maximum 3-AT inhibition level (*k*_*his*_) tolerated by *S. cerevisiae* in simulations where only the gene expression data (GED) of reaction *i* (*g*_*i*_) are considered (No GED+*g*_*i*_) or excluded (GED-*g*_*i*_). The average was normalized by the difference in the maximum *k*_*his*_ (*Δtol*) in simulations with (GED) and without (No GED) gene expression changes for all reactions. In these simulations, *k*_*ser*→*gly*_ was varied linearly with *k*_*his*_ such that when *k*_*his*_=1.0 then *k*_*ser*→*gly*_=1.0 and when *k*_*his*_=4.87×10^-1^ then *k*_*ser*→*gly*_=2.27×10^-2^. The values *k*_*his*_=1.0 and *k*_*his*_=0.0 represent no inhibition and complete inhibition, respectively. **(B)** Top ten reactions with the larger NTC magnitude. EC denotes the Enzyme Commission number. For lumped reactions (L), only the EC number for the first step is shown. **(C)** Overall gene expression changes for the ten reactions shown in **B**.

We used the model to determine the effect of gene expression changes of individual reactions on the ability of *S. cerevisiae* to grow at the dilution rate of the chemostat in the presence of 3-AT. Briefly, for each reaction, we predicted the maximum inhibition level tolerated by the *Δgcn4* and the wild-type cultures by adding or removing the gene expression changes of every reaction (one reaction at a time) and computed a normalized tolerance change (NTC) as illustrated in Figure 4A. Figure 4B shows the top ten reactions whose regulation at the gene expression level has the largest effect on the NTC (the complete list is provided in Additional file 3). The largest individual effect caused a NTC of -0.11. This result indicates that no individual reaction can be assigned a major role in the contribution to tolerance of the wild-type strain but, instead, the contribution to the tolerance was distributed among multiple reactions. Unexpectedly, neither of the two lumped reactions of the histidine synthesis pathway appeared in the top ten. The reason was that the up regulation of either of the two reactions (overall gene expression ratio of 2.1 and 2.5 for the first and second reaction, respectively) was sufficient to compensate for the inhibition of the target step (included in the first lumped reaction). However, when the gene expression changes of two reactions were removed simultaneously, the two reactions of the histidine synthesis pathway appeared in the pair with the largest NTC of 0.22. Furthermore, when the gene expression changes of three reactions were removed simultaneously, the two reactions of the histidine synthesis pathway and the lumped reaction for the synthesis of chorismate from erythrose-4-phospate appeared in the triplet with the largest NTC of 0.52. Among the 96 reactions with gene association, expression changes for 42 reactions had a positive NTC, whereas 24 reactions had a negative NTC, and the remaining had no significant effect. The sum of all individual effects was equivalent to 11% of the tolerance increase induced by all gene expression changes, whereas the sum of only the positive effects was equivalent to a 97% of the tolerance increase. This result shows that the simulated overall response required coordinated gene expression to achieve the tolerance induced by Gcn4.

Another interesting result was that the magnitude of the individual effects was not correlated with the magnitude of the gene expression changes (*ρ* = 0.06). Moreover, nine of the top ten reactions in Figure 4 had associated gene expression changes of less than two-fold. This suggests that the magnitude of gene expression changes may be a poor predictor of their importance, supporting the notion that analyses biased towards large gene expression changes may miss important insights. Note, however, that in general, small gene expression changes have more uncertainty and are more sensitive to normalization errors than large expression changes.

### Modeling the response of *S. cerevisiae* to treatment with WOAs

**Figure 5:**
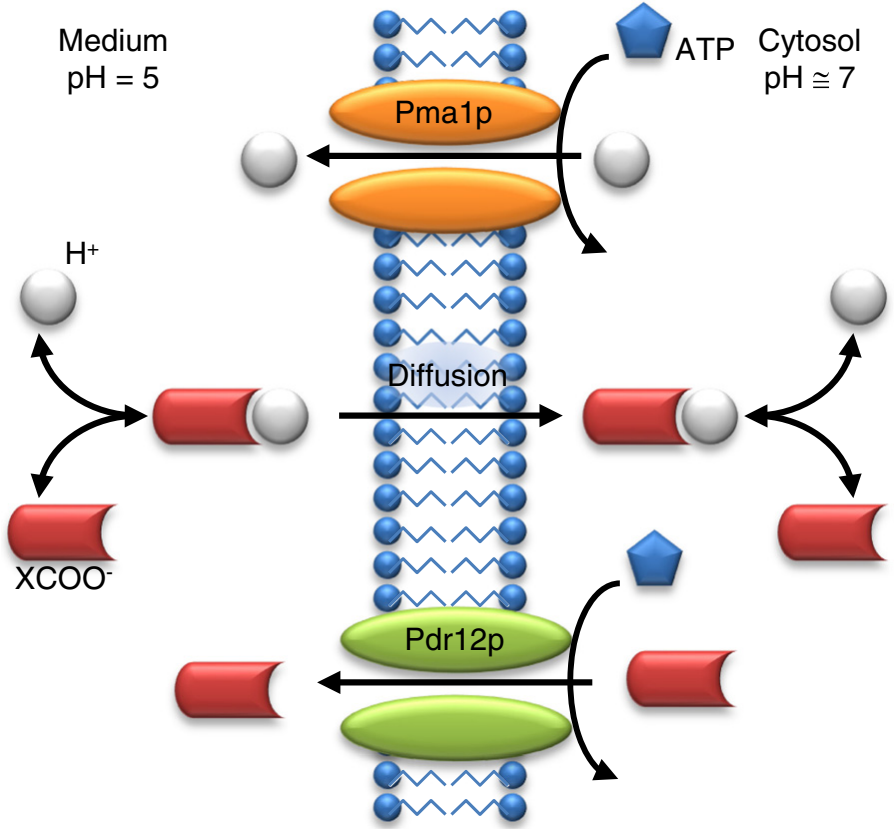
**Antimicrobial effect of weak organic acids (WOAs) and resistance mechanisms of *S. cerevisiae*.** At low extracellular pH, WOAs are mainly in their undissociated form, which can diffuse through the cellular membrane. The WOAs dissociate in the cytosol and the cell responds by upregulating transporter proteins, such as Pma1 and Pdr12, to secrete protons and carboxylate anions (XCOO^-^), respectively, to avoid toxicity.

The antimicrobial effects of WOAs, as well as the resistant mechanisms of *S. cerevisiae* to these acids, are relatively well understood [31]. Figure 5 shows the main processes involved when *S. cerevisiae* is exposed to WOAs. Briefly, at low extracellular pH, WOAs are mainly in their undissociated form, which can diffuse through the cell membrane. At a higher intracellular pH, the WOAs dissociate. *S. cerevisiae* responds by up regulating transporter proteins, such as Pma1 and Pdr12, which secrete protons and carboxylate anions, respectively, to avoid toxic accumulations. Their efflux is ATP dependent, thus reducing the available energy for biomass growth. Abbott et al. [24] investigated the transcriptional response of *S. cerevisiae* under treatment with different WOAs (acetic acid, benzoic acid, propionic acid, and sorbic acid) in anaerobic chemostat cultures. For comparison purposes, cultures were treated with one WOA at a concentration that reduced the biomass yield to 50% of the biomass yield of untreated cultures. Metabolic and gene expression profiles were obtained for these cultures and for an untreated culture, which was used as the reference condition (See Additional file 1 for details on gene expression data processing). We applied our method to construct condition-specific kinetic models to analyze the metabolic response of *S. cerevisiae* in these experiments.

#### Constructed models captured S. cerevisiae response to WOA treatment

For constructing condition-specific models, we parameterized the model using a reference flux distribution **v**^*ref*^ computed using the uptake and production rates of extracellular metabolites of the untreated culture (See Additional file 1). Subsequently, we used the gene expression ratios between the each treated culture and the reference condition **g** to parameterize the model for each treatment condition. The parameters in **p** were estimated as described in Additional File 1 and given in Table 1. We validated the constructed models by comparing the predicted metabolic responses with experimental data. Table 3 shows the predicted exchange fluxes for each WOA treatment. The agreement of the predicted fluxes with the experimental data shows that the models were able to capture the metabolic response for each WOA.

**Table 3:** Predicted metabolic responses to weak organic acid treatments

**Flux**^**a**^	**Reference**	**Treatment**							
		**Acetate**		**Benzoate**		**Propionate**		**Sorbate**	
	**Exp.**^**b**^	**Exp.**	**Sim.**	**Exp.**	**Sim.**	**Exp.**	**Sim.**	**Exp.**	**Sim.**
Glucose	6.09	12.23	12.31	12.26	12.11	12.92	12.67	12.12	11.95
CO_2_	10.45	22.54	22.58	21.80	21.67	24.09	22.89	20.76	21.55
Ethanol	9.60	21.23	21.39	20.61	20.72	21.60	21.79	20.18	20.63
Glycerol	0.79	0.54	1.56	0.96	1.43	1.00	1.56	0.83	1.28
Lactate	0.05	0.09	0.06	0.10	0.06	0.11	0.07	0.09	0.06
Acetate	0.02	−0.57	−0.58	0.08	0.11	0.03	0.12	0.02	0.11
C-recov.	99.41	95.14	98.71	95.68	98.60	96.48	99.24	93.47	98.91
Yield	0.09	0.05	0.04	0.05	0.05	0.04	0.04	0.05	0.05

^a^Fluxes from Glucose to Acetate correspond to exchange fluxes in mmol·g^-1^·h^-1^; C-recov. denotes the percentage of carbon recovered in the measured exchange fluxes and biomass (we attributed the lost carbon to pyruvate secretion); Yield denotes the production of biomass per unit of consumed substrate (g·g^-1^).

^b^ Experimental (Exp.) data taken from Abbott et al. [24]. Simulated data are denoted by Sim.

**Figure 6:**
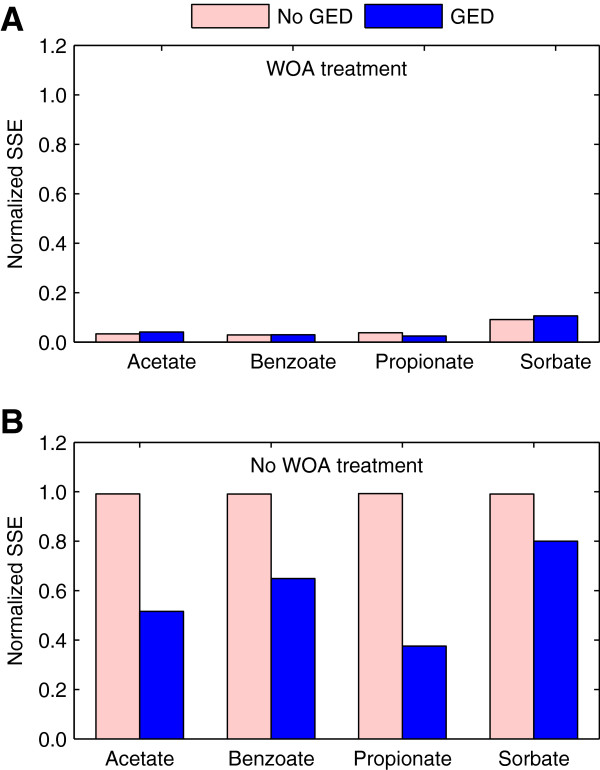
**Predicted metabolic response of *S. cerevisiae *to different weak organic acids.** Both panels show the sum of squared errors (SSE) of the predicted exchange fluxes and biomass yield normalized using the SSE between the experimental values for the reference and the corresponding treated culture. **(A)** Response of the treated cultures predicted using the corresponding gene expression data (GED) or assuming no gene expression changes (No GED). The predictions of the treated cultures using gene expression data correspond to the data in Table 3. **(B)** Predicted response of cultures under the reference condition with the expression level of the untreated culture (No GED) or with the gene expression levels of the treated cultures (GED). For simulations in **(B)**, we set the extracellular glucose and biomass concentrations to the experimental reference values.

Given their satisfactory performance, we used the models to investigate the effect of the transcriptional response on the predicted metabolic response. Thus, we compared the predicted metabolic responses to WOA treatment of cultures with and without considering the transcriptional response captured by the gene expression data (i.e., simulations with gene expression data vs. simulations without gene expression data). The results showed that gene expression changes affected metabolite concentrations and metabolic fluxes differently. Gene expression changes had a marked effect on the predicted biomass and extracellular glucose concentrations (Table 4). For all treatment conditions, the predicted concentrations using gene expression data were considerably closer to the experimental values than the predictions without using the data. Note that without considering gene expression changes, all simulations yielded identical results (except for acetic acid, which can be metabolized by *S. cerevisiae*) because all model parameters were fixed at the same values. The effect on the metabolic fluxes was less noticeable. Figure 6A shows the normalized SSE of the predicted exchange fluxes and biomass yield with respect to the experimental data. Interestingly, the accuracy of the predictions was similar with and without the gene expression data. This result is in line with the assumption that, in terms of fluxes, the structure of the metabolic network largely determines its performance [17,25].

**Table 4:** Effect of gene expression data (GED) on the predicted concentrations under weak organic acid treatments

**Simulation**	**Treatment**			
	**Acetate**	**Benzoate**	**Propionate**	**Sorbate**
**Glucose**^**a**^				
Exp.^b^	2.1 (0.1)	1.7 (0.8)	3.4 (0.3)	0.7 (0.3)
GED	0.85	3.94	0.29	10.34
No GED	36.81	41.36	41.36	41.36
**Biomass**^**a**^				
Exp.^b^	1.13 (0.02)	1.17 (0.03)	1.06 (0.03)	1.22 (0.02)
GED	1.12	1.11	1.09	1.08
No GED	0.87	0.82	0.82	0.82

^a^ Units: extracellular glucose in mmol·L^-1^; biomass in g·L^-1^.

^b^ Standard deviation in parenthesis; experimental (Exp.) data taken from Abbott et al. [24].

We further investigated the effect of the transcriptional response by comparing the predicted metabolic response of untreated cultures while considering the gene expression levels of the untreated or the treated cultures (i.e., simulations without gene expression data vs. simulations with gene expression data). Under the reference condition, the WOA uptake rate is the corresponding diffusive uptake flux of the acetic acid produced by *S. cerevisiae*. Figure 6B shows the normalized SSE of the predicted exchange fluxes and biomass yield with respect to the experimental values for the treatment conditions. In contrast to simulations without gene expression data, which by construction simulated the reference condition and had a normalized SSE of 1.0, predictions using gene expression were closer to the experimental values of the treatment conditions. This shows that the gene expression data captured, to a certain degree, the metabolic response of *S. cerevisiae* to the WOA treatments. Taken together, these results suggest that while the network structure had a predominant role on the metabolic flux distribution, gene expression changes contributed to flux regulation and had a major effect on metabolite concentration changes.

#### Effect of gene expression changes on WOA tolerance

**Figure 7:**
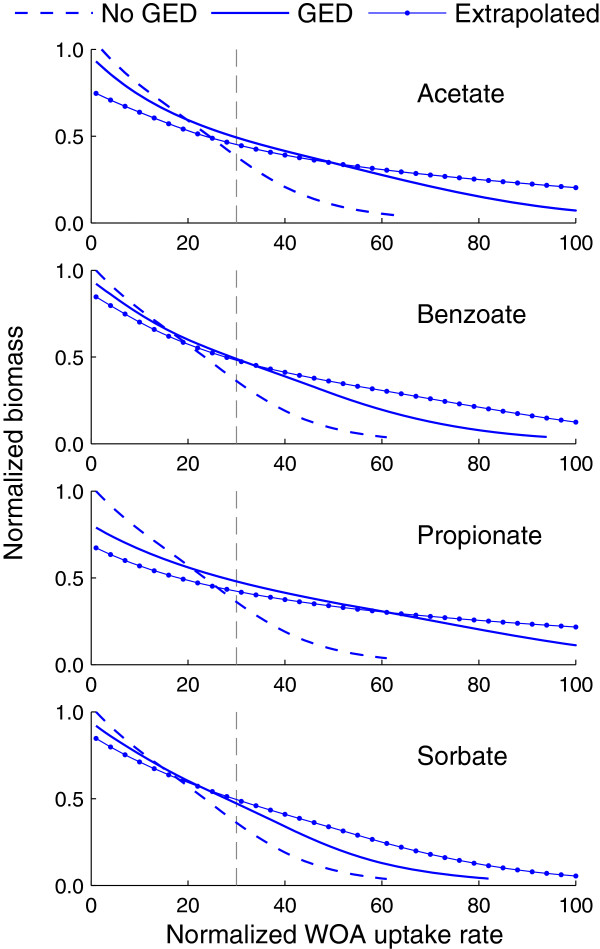
**Biomass concentration as a function of the weak organic acid (WOA) uptake rate.** The curves were constructed by using the model to simulate increasing WOA uptake rates using the experimental gene expression data (GED), assuming no gene expression changes (No GED), and by extrapolating the gene expression changes (i.e., gene expression ratios on a logarithmic scale were multiplied by 2.0). The uptake rate and biomass concentration were normalized using the uptake rate of acetic acid and biomass concentration under the reference condition, respectively. Vertical dashed lines indicate the WOA uptake rate under the treatment conditions.

We also used the constructed models to investigate the role of the transcriptional response on the tolerance of *S. cerevisiae* to WOA treatment (i.e., its ability to grow at the dilution rate of the chemostat under WOA exposure). In principle, *S. cerevisiae* should adjust its gene expression levels to better cope with these stress conditions. To probe if model predictions were in line with this premise, we predicted the biomass level as a function of the WOA uptake rate in treated cultures with and without gene expression changes. In addition, hypothesizing that the transcriptional response was graded depending on the stress intensity, we tested if, at higher WOA uptake rates, amplified gene expression changes would result in higher biomass growth than the measured gene expression changes. Thus, we also predicted the biomass levels assuming gene expression changes extrapolated from the experimental data (i.e., experimental gene expression ratios on a logarithmic scale multiplied by 2.0). Figure 7 shows the simulation results for each WOA. The models predicted that cultures with no gene expression changes produced the highest biomass level at the reference condition (i.e., normalized WOA uptake rate equal to 1.0). In contrast, simulations with the expression data had the highest biomass level (except for sorbic acid treatment) at the estimated uptake rate at which the expression data for the treated cultures were obtained (vertical dashed lines in Figure 7). Furthermore, the model predicted that cultures with extrapolated gene expression could tolerate higher WOA uptake rates, in agreement with the graded response assumption. Alternatively, this result suggests that we could predict the transcriptional response of *S. cerevisiae* to different WOA uptake rates by interpolating or extrapolating measured gene expression data. In agreement with the above premise, these simulation results suggest that the measured gene expression changes allowed *S. cerevisiae* to tolerate higher WOA uptake rates.

#### Identification of key gene expression changes for tolerance to WOA

**Figure 8:**
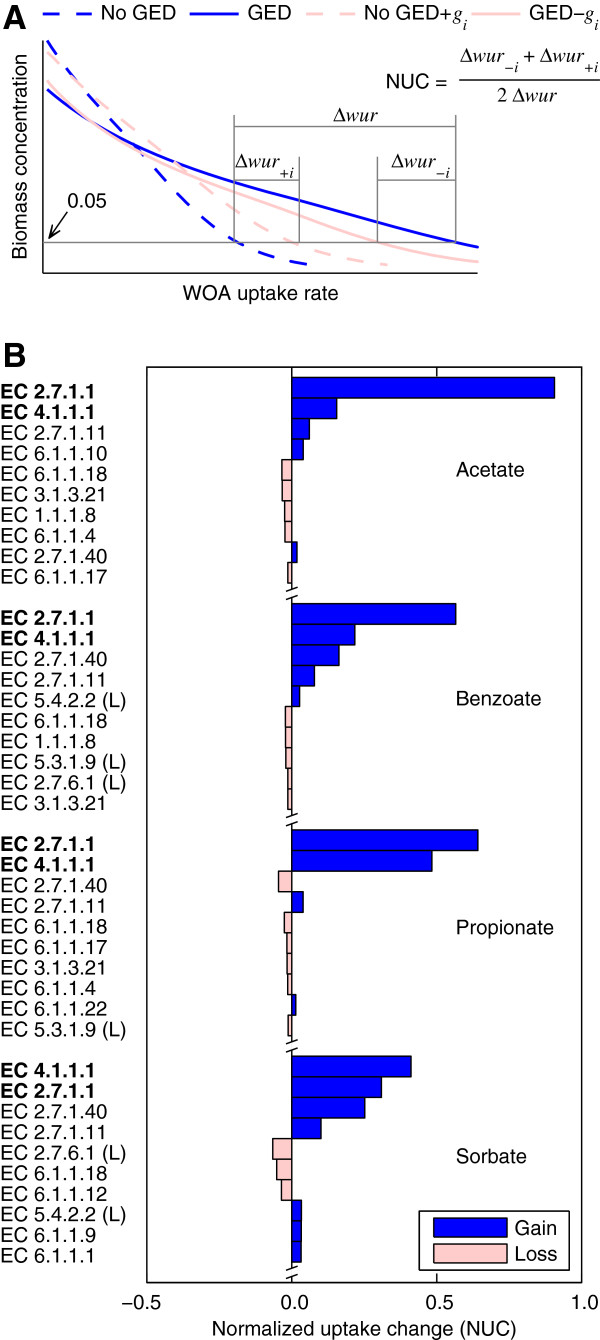
**Contribution of gene expression changes of individual reactions to weak organic acid (WOA) treatment tolerance. (A)** Definition of the metric used to compare the effects of gene expression changes of individual reactions. The normalized uptake change (NUC) for reaction *i* is defined as the average of the changes in the WOA uptake rate (*Δwur*_*+i*_ and *Δwur*_*-i*_) that reduced biomass to 5.0% of the reference value in simulations where only the gene expression data (GED) of reaction *i* (*g*_*i*_) are considered (No GED+*g*_*i*_) or excluded (GED-*g*_*i*_). The average was normalized by the difference in the WOA uptake rate that reduced the biomass to 5.0% of the reference value in simulations with (GED) and without (No GED) gene expression changes for all reactions. **(B)** Normalized uptake changes for each WOA. We only show the reactions with 10 higher contributions. EC denotes the Enzyme Commission number. The y-axis shows only the EC number for the first step of lumped reactions (L). Note that the two most influential reactions, in bold font, were EC 2.7.1.1 and EC 4.1.1.1, for all cases.

As in the simulations under histidine starvation, we used the models to determine the effect of gene expression changes associated with individual reactions on the ability of *S. cerevisiae* to grow at the dilution rate under WOA treatment. Here, we used the predicted WOA uptake rate that decreases the biomass concentration to 5.0% of the biomass of the untreated culture as a measure of tolerance as illustrated in Figure 8A. Figure 8B shows the changes in the tolerated WOA uptake rate resulting from gene expression changes associated with individual reactions. For all treatment conditions, the two most influential gene expression changes were those associated with uptake and phosphorylation of glucose (Enzyme Commission (EC) 2.7.1.1) and the decarboxylation of pyruvate to acetaldehyde (EC 4.1.1.1). Moreover, most of the increase in tolerance to the WOAs could be attributed to these two reactions, in contrast to the results under histidine starvation where the response was distributed among multiple reactions. Notably, the overall gene expression changes of these reactions were of relatively small magnitude in half of the cases (Table 5). To put this in perspective, when the reactions with gene associations (96 out of 125 reactions in the model) were sorted by descending order of magnitude of their overall gene expression changes, reaction EC 4.1.1.1 ranked below 10 in three out of the four cases. Note that we did not include in the analysis the gene expression changes associated with the efflux processes of protons and carboxylic anions [31]. Besides the two most influential reactions, only a few others had a significant individual contribution to the predicted tolerance. This result is in agreement with the “sloppiness” property, in the sense that a network’s function (growth under WOA treatment in this case) is determined by a reduced number of parameters (in this case, gene expression changes of a few reactions).

**Table 5:** Contribution to tolerance to weak organic acid (WOA) treatment and gene expression changes of the two most important reactions

**Reaction**	**Treatment**	**NUC**^**a**^	***g***^**b **^**(ranking**^**c**^**)**
EC 2.7.1.1 (Glucose → Glucose-6P)	Acetate	0.91	7.4 (3)
	Benzoate	0.56	2.4 (3)
	Propionate	0.64	5.9 (2)
	Sorbate	0.31	1.4 (35)
EC 4.1.1.1 (Pyruvate → Acetaldehyde)	Acetate	0.16	1.4 (28)
	Benzoate	0.22	1.4 (21)
	Propionate	0.48	4.1 (6)
	Sorbate	0.41	1.6 (15)

^a^ NUC: normalized uptake change. See Figure 8A for the definition of NUC.

^b^ Overall gene expression changes associated with the reaction computed from the gene expression data taken from Abbott et al. [24].

^c^ Ranking among the 96 reactions associated with at least one gene, sorted by decreasing level of gene expression changes (absolute value of the log_2_ expression ratio).

### Sensitivity and robustness of model predictions

An advantage of our method is that it has a small number of general fitting parameters: *α*, *β*, and *γ*. We investigated the effect of these fitting parameters on model predictions by simulating the model for different values of these parameters. For the WOA treatment experiments, we determined the normalized SSE between the predicted and experimental values of the exchange fluxes and biomass yield as a function of each parameter. The analysis showed that the predicted fluxes were robust with respect to changes in these parameters (see Additional File 1). The simulations of the histidine starvation experiments were relatively robust to variations in these parameters around the values reported in Table 1, as shown in Additional File 1.

We also investigated if the method was sensitive to the input gene expression data or if the results could be obtained with arbitrary data. Briefly, we simulated the models with data generated by randomly shuffling the original expression data. The simulation results showed that it is unlikely that similar results could be obtained using random gene expression data (see Additional File 1). Moreover, uncertainty propagation analysis showed that the method is robust with respect to experimental noise in the gene expression data and flux distributions (Additional File 1).

## Discussion

Long-standing barriers impeding the construction of large-scale kinetic models of metabolism are being overcome with the help of developments in high-throughput technologies and computational analyses. Modelers are now faced with the challenge of integrating the increasingly available building blocks to create coherent mathematical representations of biological systems. Here, we presented our efforts to develop a modeling framework for constructing large-scale kinetic models that mechanistically link transcriptional regulation and metabolism. This allowed us to gain understanding of complex physiological relations from fluxome, metabolome, and gene expression data. We demonstrated the ability of our method to capture these relations, its flexibility to simulate different experiments, and its robustness with respect to modeling approximations and data uncertainty by analyzing the response of *S. cerevisiae* under different stress conditions. Importantly, our approach can be applied to other organisms of medical and industrial relevance (or cell types in multi-cellular organisms) for which a metabolic network reconstruction, metabolic flux measurements, and gene expression data are available for the conditions of interest.

### The method provides efficient solutions to large-scale modeling challenges

One of the major challenges in constructing large-scale kinetic models is the definition of appropriate reaction rate expressions. Instead of defining mechanistic reaction rate expressions on a case-by-case basis, some approaches streamline this process by relying on generic expressions to translate a metabolic network into a kinetic model in an automated or semi-automated fashion. Different general forms have been proposed, such as log-linear kinetics [32], Michaelis-Menten-type kinetics [33], “convenience” kinetics [19], or GMA kinetics [23]. GMA kinetics are used, for example, in ensemble modeling [20] and mass action stoichiometric simulation (MASS) models [21]. In ensemble modeling and MASS models, the enzymatic reactions are decomposed into their elementary steps, and each step is then modeled using mass action kinetics. The decomposition increases the resolution of the model, preserves enzyme saturation behavior, and simplifies the parameter estimation problem, but at the price of considerably increasing the size of the model (i.e., the number of dynamic variables and model parameters) and the amount of data required to estimate parameter values. In contrast, we used a special case of GMA kinetics that requires a minimal number of parameters, which can be obtained directly from available experimental data (see Methods and Additional File 1). Moreover, enzymatic reactions were not decomposed into elementary steps to avoid increasing the size of the model.

Another challenge is the determination of model parameter values. The difficulty in solving this problem is linked to the form of the kinetic expressions and to the availability of experimental data. If experimental data are not available, approaches such as log-linear kinetics and “convenience” kinetics require mining the literature for parameter values, which (aside from the skepticism about the validity of combining parameter values from different conditions to simulate a specific experiment) could be impractical for large-scale models. Approaches using GMA kinetics partially avoid literature mining. In these approaches, such as MASS modeling [21], thermodynamic information collected from the literature (e.g., equilibrium constants, Gibbs free energies, etc.) is combined with experimentally determined metabolite and/or enzyme concentrations and flux distributions to estimate the remaining model parameters (i.e., the rate constants). For the common case of incomplete data, the missing information is approximated to “typical” values or is randomly generated to create an ensemble of models that are screened for models that agree with experimental observations [20]. Based on the “sloppiness” property, we would expect that models parameterized using “typical” values will perform reasonable well. However, the typical values generally fall within relatively wide ranges, making the selection of parameter values to simulate a particular condition a non-trivial task. In contrast, the rate expressions we used enabled us to readily obtain the bulk of the model parameters (221 out of 227) directly from available experimental data (i.e., flux distributions and gene expression; see Methods and Additional File 1). Moreover, we circumvented mining the literature or using randomly generated values for thermodynamic parameters by assuming a single parameter (*β*) for relating the forward and backward reaction rates to the overall rate for all reversible reactions. This crude approximation, inspired in part by the “sloppiness” property of biological systems, worked surprisingly well for the examples studied here. Our method performed well even if the uptake and production rates of extracellular metabolites were the only metabolic data available, as demonstrated in the analysis of *S. cerevisiae* tolerance to WOAs (see Additional File 1 for simulations using only uptake and production rates of extracellular metabolites under histidine starvation).

An additional attribute of our method is the use of gene expression data to parameterize the model to simulate different conditions, an element that has been used in constraint-based approaches to create context-specific models [7-10], but which has not been fully exploited in other kinetic modeling approaches. An exception is the work by Bruck et al. [34], in which gene expression was integrated with a kinetic model of *S. cerevisiae* glycolysis based on a mechanistic model developed by Teusink et al. [35]. However, Bruck et al. [34] estimated a subset of 31 parameters to fit the model to data from all conditions they simulated and did not present simulations without the gene expression data, preventing an assessment of the contribution of gene expression changes. In contrast, our models were able to simulate metabolic responses with a smaller subset of fitting parameters and our analysis showed the important role of gene expression on model predictions. Note that requiring gene expression data in order to simulate other conditions could also be considered a weakness, but no other model includes the prediction of protein/gene expression changes for the systems of the size of the network we analyzed.

### Constructed models generated biological insights

We demonstrated that the constructed models were able to integrate transcriptional and metabolic responses to produce insights that would have been difficult to grasp from the analysis of the individual responses. For example, in their analysis of *S. cerevisiae* response to WOAs, Abbott et al. [24] identified differentially expressed genes as those with an expression change larger than two-fold and a false discovery rate lower than 0.5%. With these criteria, they found hundreds of differentially expressed genes under each treatment condition, but only 14 genes that were upregulated under all treatment conditions. Therefore, they concluded that the generic (i.e., common to all treatments) transcriptional response to WOAs was minimal and suggested that more relevance should be given to the specific responses to the specific treatment conditions. We agree that attention should be paid to the specific responses, but our analysis also suggests that the generic response, despite involving a few genes, is a major factor contributing to WOA tolerance. Based on our simulation results, we hypothesize that *S. cerevisiae* tightly regulates the expression levels of two reactions (glucose uptake-phosphorylation and decarboxylation of pyruvate to acetaldehyde) to increase the tolerance under all treatment conditions. Firstly, this generic response was not identified in the Abbott et al. [24] analysis because the gene expression changes for these reactions did not meet their criteria for differentially expressed genes (see Additional File 1). Secondly, we estimated that regulating these two reactions accounted for most of the increase in tolerance to WOAs (Figure 8 and Table 5). If correct, this hypothesis implies that *S. cerevisiae* has a generic response to WOAs that is critical for the adaptation to these stressors.

Identification of important reactions in a metabolic network has been one of the major goals of several model-based approaches. For example, Kummel et al. [36] developed a thermodynamics-based method to identify regulated reactions, assuming that reactions far from equilibrium are more likely to be regulated. In contrast with our method, their approach does not use any kinetic information but requires thermodynamic and metabolome data. In another example, Smallbone et al. [22] combined log-linear kinetics with metabolic control analysis [37,38] to identify reactions exerting the most control over biomass production in a genome-scale metabolic network of *S. cerevisiae*. Similar to these efforts, our method was able to identify important regulated reactions under specific conditions. However, our method also provided mechanistic insights into how the cell regulates such reactions through transcriptional regulation and how this response is reflected in its phenotype.

In another effort to link the regulatory and metabolic responses, Moxley et al. [25] proposed a hybrid approach to predict changes in metabolic fluxes using gene expression changes. Their approach was based on the assumption that gene expression changes and fluxes are more correlated in pathways with fewer metabolite-enzyme interactions (metabolite-enzyme interactions exist between an enzyme and metabolites that regulate its activity). Thus, their approach combined a metabolic network model with a metabolite-enzyme interaction network. Using this approach, they predicted flux changes that had a relatively high correlation (*ρ* = 0.80) with the experimentally estimated flux changes for a subset of reactions. For the same subset, our model predictions showed a considerably higher correlation (*ρ* = 0.96). Moreover, our method required less information because knowledge of the metabolite-enzyme interaction network is not needed. Interestingly, their predictions, using only the metabolic network model (without considering metabolite-enzyme interactions), had a similar *ρ* of approximately 0.75, reflecting the major contribution of the network structure to its function. In terms of biological insights, they observed a redistribution of the glycine synthesis fluxes. They proposed that the increase in glycine production from threonine is mediated by the increased expression of the associated genes, but they do not fully explain why the flux from serine to glycine decreased. Our analysis led to the plausible explanation that the decrease in the flux from serine to glycine could have been caused by the decrease of tetrahydrofolate, which, in turn, could have been caused by off-target inhibitions of 3-AT. In addition, and in contrast with their approach, our method also predicted concentration changes. In fact, we are unaware of other modeling efforts with similar scope that produce similar levels of accuracy, using condition-specific data directly as model parameters and using only five fitting parameters.

An additional conjecture about the use of gene expression changes to parameterize protein activity changes can be derived from our simulation results. We omitted post-translational and other regulatory mechanisms and yet the model predictions were consistent with experimental data. This suggests that, for the metabolic network and the experiments considered here, transcriptional regulation was the main mechanism that regulated the response at the system level. Moreover, the accuracy of the model predictions suggests that gene expression changes were a good approximation for protein level changes, in agreement with experimental observations [27,39].

### Further developments

The proposed method does not need knowledge of the absolute values of metabolite concentrations for steady-state simulations, but these are required for analysis of transient behavior. Developments in analytical techniques have increased the accuracy and scope of metabolite concentration measurements. However, such data are still generally incomplete and, thus, missing data must be estimated or assumed. Note that the requirement of metabolite concentrations to describe dynamic behavior is common to similar modeling approaches. Thus, it remains to be investigated how the proposed modeling framework performs in describing dynamic and transient properties associated with metabolic processes.

The models constructed with the proposed method present some limitations. For example, the generic rate expressions may be poor approximations for some reactions or may miss important allosteric regulations (e.g., feedback loops) and other factors that have an effect on protein activity and abundance (e.g., post-translational modifications). Lumping sequential reactions reduced the size of the model. However, in our approach, the rate expressions for lumped reactions are only an approximation to the sequence of individual reactions. In the experiments we analyzed, the final results were not sensitive to our somewhat arbitrary parameter choice *m*_*i*_ and *β.* This may not be always the case and estimating more accurate parameters values may be necessary. As for any method, identifying and correcting modeling errors is a painstaking task. This could be especially true for automated model generation. Procedures to address this problem in a systematic way need to be developed. Furthermore, our method needs to be tested to determine whether it can be applied to genome-scale metabolic networks. Such application could be problematic because of the higher uncertainty of lowly expressed genes and small metabolic fluxes, the buildup of approximation errors, and numerical challenges to solve the model. Regarding its scope, the proposed method is limited to gene expression and metabolism. Although it enables a deeper, mechanistic analysis of these processes, further developments to include other cellular processes (e.g., signal transduction, cell division, etc.) would greatly enhance the modeling framework.

## Conclusions

In summary, we investigated how gene expression changes induce metabolic responses when cells adapt to a stressful condition. For this purpose, we developed a modeling framework for constructing and simulating large-scale kinetic models that provided a mechanistic link between transcriptional regulation and cellular metabolism. Analysis of the response of *S. cerevisiae* to treatment with WOA and under histidine starvation generated several insights and testable hypotheses: *1*) 3-AT also inhibits the synthesis of tetrahydrofolate; *2*) *S. cerevisiae* has an important generic response to WOA, involving glucose uptake and decarboxylation of pyruvate to acetaldehyde; *3*) the contribution to tolerance to 3-AT is distributed among several reactions while the contribution to tolerance to WOA is mainly concentrated in two reactions; *4*) the magnitude of gene expression changes was not correlated with the magnitude of their effect on the overall response. Taken together, these results suggest that the proposed framework is able to dissect different “omics” data to determine important features of the transcriptional-metabolic response of *S. cerevisiae*.

## Competing interests

The authors declare that they have no competing interests.

## Author contributions

All authors contributed to the design of the research. FGV performed the computational implementations. FGV and AW wrote the article, which was edited by JR. All authors read and approved the final manuscript.

## Supplementary Material

Additional file 1 Supplementary materials (pdf file): detailed information about the method for constructing large-scale kinetic models, fitting parameters, off-targets of 3-AT, and expression profiles of important genes under weak organic acid treatment. Click here for file

Additional file 2 Kinetic model (Zip file): MATLAB scripts for simulating the kinetic model and a XML file with the model in SBML format). Click here for file

Additional file 3 Metabolic network (Excel file): detailed description of the reactions in the metabolic network and lists of the ranked reactions according to its effect on reducing inconsistencies and on tolerance to 3-AT. Click here for file
