# Supplementary material for: Bridging the gap between gene expression and metabolic phenotype via kinetic models
Source: BMC Syst Biol. 2013 Jul 22;7:63. doi: 10.1186/1752-0509-7-63 (PMC3733621; doi:10.1186/1752-0509-7-63)
Supplement: Additional file 1 — Supplementary materials (pdf file): detailed information about the method for constructing large-scale kinetic models, fitting parameters, off-targets of 3-AT, and expression profiles of important genes under weak organic acid treatment. [file 1752-0509-7-63-S1.pdf]

Supplementary Materials

Bridging the Gap between Gene Expression and Metabolic Phenotype via Kinetic Models

Francisco G. Vital-Lopez, Anders Wallqvist, and Jaques Reifman

DoD Biotechnology High Performance Computing Software Applications Institute, Telemedicine and Advanced Technology Research Center, U.S. Army Medical Research and Materiel Command, Ft. Detrick, MD 21702, USA

Table of contents

1. Method for constructing large-scale kinetic models

Derivation of generic kinetic expressions

Thermodynamic constraints on β

Rate expressions for lumped reactions

Overall gene expression change for reactions associated with multiple genes

Method for computing a reference flux distribution from measured uptake/production rates

2. Estimation of fitting parameters

Estimation of α, β, and δ

Parameters specific for the analysis of *S. cerevisiae*'s response to WOA treatment

Parameters specific for the analysis of *S. cerevisiae*'s response to histidine starvation

3. Processing of gene expression data

4. Experimental flux distributions for *Δgcn4* and wild-type cultures

5. Simulations under histidine starvation using different values for the constants m_i

6. Simulations under histidine starvation without gene expression data

7. Identification of candidate off-targets of 3-aminotriazole

8. Sensitivity analysis of fitting parameters

9. Expression profiles of the two most determinant reactions for tolerance to WOAs

10. References

1. Method for constructing large-scale kinetic models

Derivation of generic kinetic expressions

Consider a set of metabolites that are transformed in a single irreversible reaction:

$$\sum_i a_i A_i \rightarrow \sum_j b_j B_j, \tag{S1}$$

where a_i and b_j denote the stoichiometric coefficients of species A_i and B_j, respectively. We modeled the rate of this reaction with the expression:

$$r = k[\bar{p}]\frac{\prod_i [\bar{A}_i]^{m_i}}{\prod_j [\bar{B}_j]^{m_j}}, \tag{S2}$$

where r denotes the reaction rate, k is the rate constant, p is the overall level of the proteins associated with the reaction, and the square brackets and the upper bars denote absolute metabolite concentrations. The constants m_i (m_j) are set to 2.0 if a_i (a_j) is 2.0, or to 1.0 otherwise. The purpose of including product inhibition was to enable downstream reactions to have an effect on the flux through a pathway via a direct feedback mechanism. We normalized the overall protein level and metabolite concentrations with respect to a reference condition:

$$g = \frac{[\bar{p}]}{[\bar{p}^{ref}]}, \tag{S3}$$

$$[A_i] = \frac{[\bar{A}_i]}{[\bar{A}_i^{ref}]}, \tag{S4}$$

where g denotes the normalized overall protein level and $[A_i]$ represents the normalized metabolite concentration. The reaction rate can be expressed as,

$$r = v^{ref} g \frac{\prod_i [A_i]^{m_i}}{\prod_j [B_j]^{m_j}}, \tag{S5}$$

where all the parameters of the expression are lumped into a single parameter that corresponds to the flux of the reaction at the reference condition,

$$v^{ref} = k[\bar{p}^{ref}]\frac{\prod_i [\bar{A}_i^{ref}]^{m_i}}{\prod_j [\bar{B}_j^{ref}]^{m_j}}. \quad \text{(S6)}$$

Similarly, for a single reversible reaction:

$$\sum_i a_i A_i \leftrightarrow \sum_j b_j B_j. \quad \text{(S7)}$$

After normalizing the protein levels and metabolite concentrations, the reaction rate can be written as,

$$r = g\left(v^f \prod_i [A_i]^{m_i} - v^b \prod_j [B_j]^{m_j}\right), \quad \text{(S8)}$$

where v^f and v^b are determined as follows:

$$v^f = v^{ref}\beta \text{ and } v^b = v^{ref}(\beta - 1), \text{ if } v^{ref} > 0, \quad \text{(S9)}$$

$$v^f = v^{ref}(\beta - 1) \text{ and } v^b = v^{ref}\beta, \text{ if } v^{ref} < 0, \quad \text{(S10)}$$

where β is a fitting parameter.

Thermodynamic constraints on β

By definition, β is constrained to be larger than 1.0. In addition, β must satisfy thermodynamic constrains for parallel pathways: the overall equilibrium constant should be the same for all parallel pathways. The relation between β and the equilibrium constants is given by:

$$\beta = \frac{K_{eq}/Q}{K_{eq}/Q - 1}, \text{ if } v^{ref} > 0, \quad \text{(S11)}$$

$$\beta = \frac{1}{1 - K_{eq}/Q}, \text{ if } v^{ref} < 0, \quad \text{(S12)}$$

$$Q = \prod_i [\bar{A}_i^{ref}] \Big/ \prod_j [\bar{B}_j^{ref}]. \quad \text{(S13)}$$

We have two cases of parallel pathways in the metabolic network that impose the following constraints:

$$K_{eq,25}K_{eq,32}K_{eq,33} = K_{eq,30}K_{eq,31}, \tag{S14}$$

$$K_{eq,29}K_{eq,55} = K_{eq,54}. \tag{S15}$$

Substituting the equilibrium constants as a function of β for the flux distribution of the *Δgnc4* mutant under 3-aminotriazole treatment, we obtained the constraints:

$$\frac{\beta_{32}}{\beta_{32}-1}\frac{\beta_{33}}{\beta_{33}-1} = \frac{\beta_{25}}{\beta_{25}-1}\frac{\beta_{30}}{\beta_{30}-1}\frac{\beta_{31}}{\beta_{31}-1}, \tag{S16}$$

$$\frac{\beta_{55}}{\beta_{55}-1} = \frac{\beta_{29}}{\beta_{29}-1}\frac{\beta_{54}}{\beta_{54}-1}. \tag{S17}$$

By arbitrarily setting $\beta_{55} = \beta_{32} = \beta_{33} = \beta$, $\beta_{29} = \beta_{54}$, and $\beta_{25} = \beta_{30} = \beta_{31}$,we finally obtained:

$$\beta_{25} = \beta_{30} = \beta_{31} = \frac{\left(\beta/(\beta-1)\right)^{2/3}}{\left(\beta/(\beta-1)\right)^{2/3}-1}, \tag{S18}$$

$$\beta_{29} = \beta_{54} = \frac{\left(\beta/(\beta-1)\right)^{1/2}}{\left(\beta/(\beta-1)\right)^{1/2}-1}. \tag{S19}$$

Similarly, for the flux distribution of the reference culture for the weak organic acid treatment experiments, we obtained the constraints:

$$\beta_{54} = \beta_{32} = \beta_{33} = \beta \tag{S20}$$

$$\beta_{25} = \beta_{30} = \beta_{31} = \frac{\left(\beta/(\beta-1)\right)^{2/3}}{\left(\beta/(\beta-1)\right)^{2/3}-1}, \tag{S21}$$

$$\beta_{29} = \beta_{55} = \frac{\left(\beta/(\beta-1)\right)^{1/2}}{\left(\beta/(\beta-1)\right)^{1/2}-1}. \tag{S22}$$

Rate expressions for lumped reactions

To simplify the derivation, consider the sequence of two irreversible reactions:

$$\sum_i a_i A_i \xrightarrow{r_1} \sum_j b_j B_j \xrightarrow{r_2} \sum_k d_k D_k, \tag{S23}$$

where d_k denotes the stoichiometric coefficients of species D_k. The rates of these two reactions are described by the expressions:

$$r_1 = v_1^{ref} g_1 \frac{\prod_i [A_i]^{m_i}}{\prod_j [B_j]^{m_j}}, \tag{S24}$$

$$r_2 = v_2^{ref} g_2 \frac{\prod_j [B_j]^{m_j}}{\prod_k [D_k]^{m_k}}. \tag{S25}$$

At steady-state,

$$r = r_1 = r_2, \tag{S26}$$

$$v = v_1^{ref} = v_2^{ref}, \tag{S27}$$

where r denotes the rate of the lumped reaction and v represents the flux through the reactions. Equations (S24) and (S25) can be solved for the product of concentrations of B_j:

$$\prod_j [B_j]^{m_j} = \left(\frac{v_1^{ref} g_1 \prod_i [A_i]^{m_i} \prod_k [D_k]^{m_k}}{v_2^{ref} g_2} \right)^{1/2}. \tag{S28}$$

Substituting Eq. (S28) into Eq. (S24) and using Eq. (S26) and Eq. (S27) gives,

$$r = v^{ref} \left(\frac{g_1 g_2 \prod_i [A_i]^{m_i}}{\prod_k [D_k]^{m_k}} \right)^{1/2}. \tag{S29}$$

This can be generalized for *n* sequential irreversible reactions between substrates A_i and products D_k as,

$$r = v^{ref}\left(\frac{\prod_h g_h \prod_i [A_i]^{m_i}}{\prod_k [D_k]^{m_j}}\right)^{1/n}. \tag{S30}$$

For a sequence of reversible reactions:

$$\sum_i a_i A_i \overset{r_1}{\leftrightarrow} \sum_j b_j B_j \overset{r_2}{\leftrightarrow} \sum_k d_k D_k. \tag{S31}$$

The rates of these two reactions are described by the expressions:

$$r_1 = g_1\left(v_1^f \prod_i [A_i]^{m_i} - v_1^b \prod_j [B_j]^{m_j}\right), \tag{S32}$$

$$r_2 = g_2\left(v_2^f \prod_j [B_j]^{m_i} - v_2^b \prod_k [D_k]^{m_j}\right). \tag{S33}$$

Assuming that under steady-state conditions:

$$v^f = v_1^f = v_2^f, \tag{S34}$$

$$v^b = v_1^b = v_2^b, \tag{S35}$$

after algebraic manipulations of Eq. (S32) to Eq. (S35), the rate of the lumped reaction is given by:

$$r = \frac{g_1 g_2 \beta}{g_1(\beta-1) + g_2\beta} v^f \prod_i [A_i]^{m_i} - \frac{g_1 g_2(\beta-1)}{g_1(\beta-1) + g_2\beta} v^b \prod_k [D_k]^{m_k}. \tag{S36}$$

To further simplify the expressions, we used the following heuristic approximations:

$$\frac{g_1 g_2 \beta}{g_1(\beta-1) + g_2\beta} \cong \frac{g_1 g_2(\beta-1)}{g_1(\beta-1) + g_2\beta} \cong (g_1 g_2)^{1/2}, \tag{S37}$$

and the rate expression for the lumped reversible reaction:

$$r = (g_1 g_2)^{1/2}\left(v^f \prod_i [A_i]^{m_i} - v^b \prod_k [D_k]^{m_k}\right). \tag{S38}$$

This approximate expression is close to the exact expression when β is much larger than 1.0 and g_1 and g_2 are similar (see Figure 1), and allows us to use the same expression to compute the overall gene expression changes for both reversible and irreversible reactions.

For sequential reactions involving irreversible and reversible reactions, the expressions became too convoluted. Therefore, we approximated their reaction rate as,

$$r = v^{ref}\left(\prod_h g_h\right)^{1/n}\left(\frac{\prod_i [A_i]^{m_i}}{\prod_k [D_k]^{m_k}}\right)^{1/\gamma}, \tag{S39}$$

where γ is the number of irreversible steps.

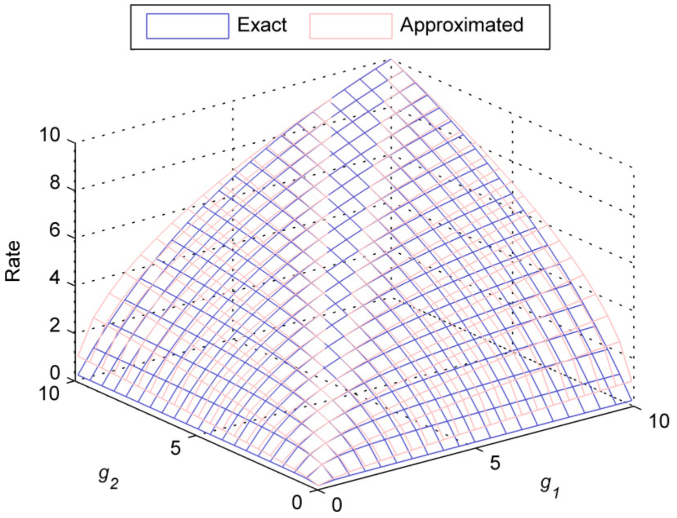

Figure 1. Exact and approximate reaction rates for lumped reversible reactions. The reaction rates were computed for different values of g_1 and g_2, assuming that the overall reference flux and all concentrations were equal to 1.0.

Overall gene expression change for reactions associated with multiple genes

Consider a single-step reaction associated with *n* genes. There are two basic cases for computing the overall gene expression change:

1. All genes are required for the reaction to occur (e.g., the genes code for subunits of an enzyme):

$$g = \left(\prod_i g_i\right)^{1/n}. \tag{S40}$$

2. Genes code for independent isoenzymes:

$$g = \frac{1}{\sum_i w_i} \sum_i w_i g_i, \tag{S41}$$

where w_i is a weight given to gene *i*. In the examples shown in the main text, the weights were a function of the expression intensity (h_i) of each gene:

$$w_i = 1 + \frac{1}{n} \sum_i \log_2(h_i). \tag{S42}$$

Thus, highly expressed genes were given more weight than lowly expressed genes.

For more complicated reaction-gene associations, the overall gene expression change was computed by applying the appropriate mean (arithmetic or geometric) in a recursive way. For example, for a lumped reaction, first the overall gene expression change for each step was computed. Then, the overall expression change for the lumped reaction was computed using the geometric mean, because all steps of the lumped reaction were required.

Method to compute a reference flux distribution from measured uptake/production rates

A reference flux distribution that satisfies the stoichiometric constrains and the measured uptake and production rates of extracellular metabolites can be obtained with the following four-step procedure:

1. Compute the lower and upper bound of every reaction in the network. The lower bound was computed by solving the optimization problem:

$$
\begin{aligned}
&\min v_i \\
&\text{s.t.} \\
&\mathbf{S} \cdot \mathbf{v} = \mathbf{0} \\
&v_i \geq 0 \quad \forall i \in IR \\
&v_i = v_i^{exp} \quad \forall i \in X
\end{aligned}
\tag{S43}
$$

where v_i is the flux of reaction *i*, $\mathbf{S}$ is the stoichiometric matrix, $\mathbf{v}$ is a vector of elements v_i, *IR* is the set of irreversible reactions, v_i^{exp} is the experimental value of reaction i, and *X* is the set of measured reactions. This problem determines the lower bound of reaction i. To compute the upper bound the objective function is maximize instead.

2. Some of the reactions may have an unbounded lower bound, upper bound, or both. This generally occurs for the presence of cycles in the metabolic network. To compute meaningful lower and upper bounds for an unbounded reaction, we constrained the flux of one of the reactions in a cycle to zero and computed the lower and upper limits for the rest of the reactions in the cycle. We took the lowest and largest values of each reaction in the broken cycle estimations as the lower and upper bounds for the unbounded reactions, respectively.

3. Compute a flux distribution that minimizes the distance to the center of the box defined by the lower (v_i^l) and upper (v_i^u) bounds of the fluxes and that satisfies the constraints of the problem in Step 1:

$$
\begin{aligned}
&\min \sum_i \left| v_i - (v_i^l + v_i^u)/2 \right| \\
&\text{s.t.} \\
&\mathbf{S} \cdot \mathbf{v} = \mathbf{0} \\
&v_i \geq 0 \quad \forall i \in IR \\
&v_i = v_i^{exp} \quad \forall i \in X
\end{aligned}
\tag{S44}
$$

4. The solution of the problem in Step 3 may have some of the reactions with zero flux, even if their lower or upper bounds are non-zero. However, we assumed that if the enzyme(s) and substrate(s) of a given reaction are present, then the reaction must have a non-zero flux. To compute a non-zero flux for such reactions we solved the following problem:

$$
\begin{aligned}
&\min \sum\nolimits_{i \in Z} \left| v_i - (v_i^l + v_i^u)/2 \right| \\
&\text{s.t.} \\
&\mathbf{S} \cdot \mathbf{v} = \mathbf{0} \\
&v_i \geq 0 \quad \forall i \in IR \\
&v_i = v_i^{exp} \quad \forall i \in X \\
&\sum\nolimits_i \left| v_i - (v_i^l + v_i^u)/2 \right| \leq z_0 + L
\end{aligned}
\tag{S45}
$$

where Z is the set of reactions with zero flux, z_0 is the objective function value for the solution of the problem in Step 3, and L is a slack parameter. This problem is solved iteratively until all possible reactions have a non-zero flux. Then, the reference flux distribution is computed from all solutions:

$$
\mathbf{v}^{ref} = \frac{1}{m} \sum\nolimits_j \mathbf{v}_j, \tag{S46}
$$

where m is the number of solutions $\mathbf{v}_j$.

We tested the method to compute a reference flux distribution using only uptake and production rates from Moxley et al. [1]. Figure 2 shows the predicted flux distribution plotted against the experimental flux distribution for the *gcn4*-knockout mutant culture. The Pearson's correlation coefficient between the predicted and experimental fluxes was 0.93. Figure 3 shows the flux distribution and concentrations of the free amino acids of the wild-type cultured predicted using the estimated flux distribution for the *gnc4*-knockout mutant culture. The correlation coefficient between for the flux distribution and the concentration changes of free amino acids was 0.93 and 0.90, respectively. This shows that the estimated flux distribution worked well to parameterize the kinetic model.

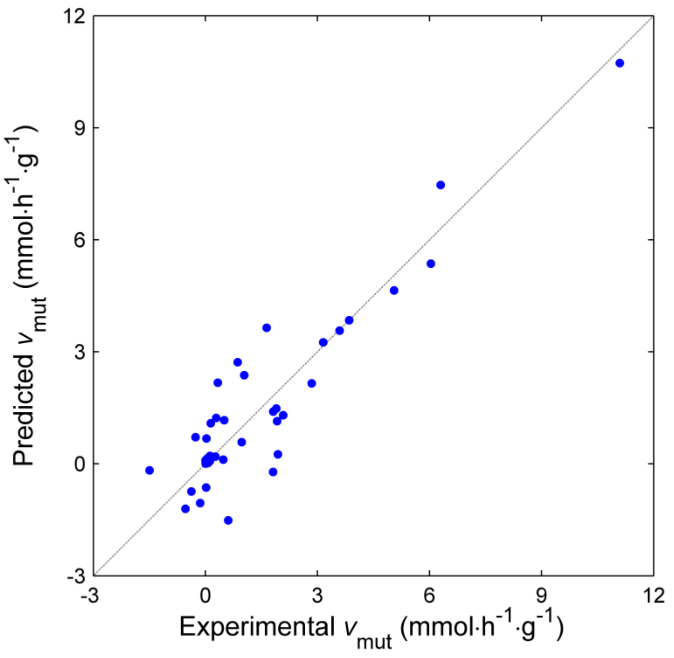

Figure 2. Predicted flux distribution for the *Δgcn4* culture using only its uptake and production rate measurements. Experimental data were taken from Moxley et al. [1].

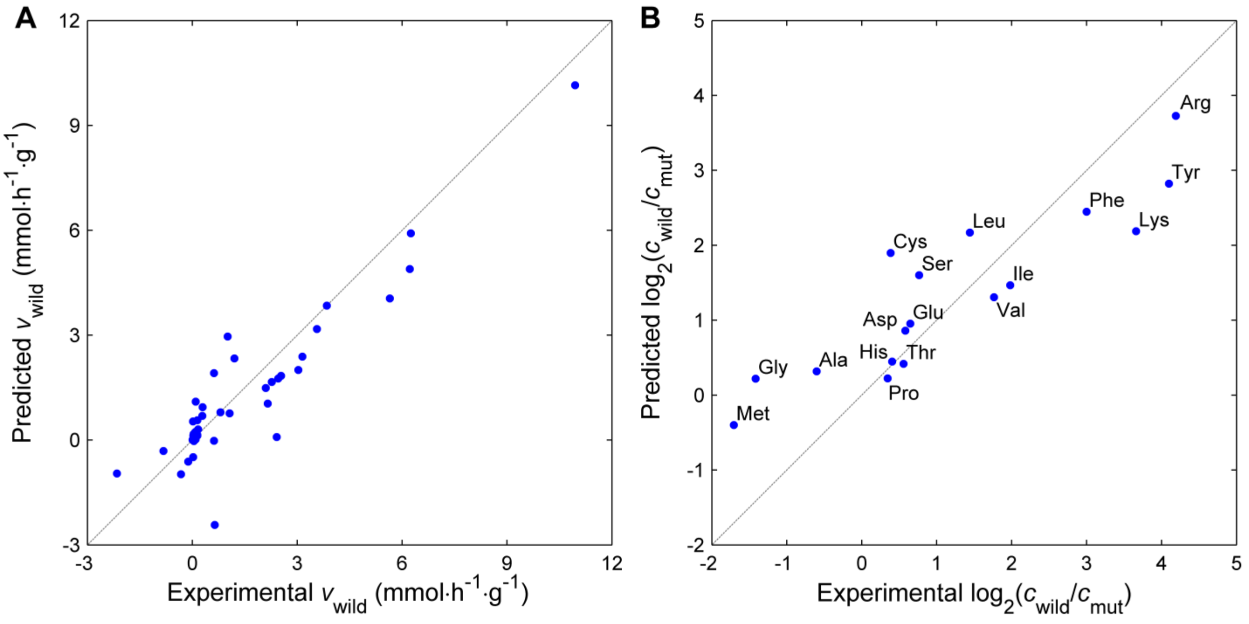

Figure 3. Predicted flux distribution and free amino acid concentrations for the wild-type culture using only uptake and production rate measurements of the *Δgcn4* culture. Experimental data were taken from Moxley et al. [1].

2. Estimation of fitting parameters

Estimation of α, β, and δ

We estimated the parameters α, β, and δ by minimizing the sum of squared error between the simulated and the experimental amino acid concentration changes. Figure 4 shows the landscape of the sum of squared errors (A-C), the Pearson's correlation coefficient (D-F) and the slope of the best fit (G-I) for the amino acid concentration changes in the space of these parameters. Note that the results are relatively robust at the neighborhood of the minimum ($\alpha = 0.10$, $\beta = 30$, and $\delta = 0.98$). Figure 4J-L shows the Pearson's correlation coefficient between the simulations and experimental measurements for the subset of fluxes used in Moxley et al. [1].

For the analysis of the response of *S. cerevisiae* to treatment with weak organic acids, we used the values $\alpha = 0.10$, and $\beta = 30$, but we used $\delta = 1$.

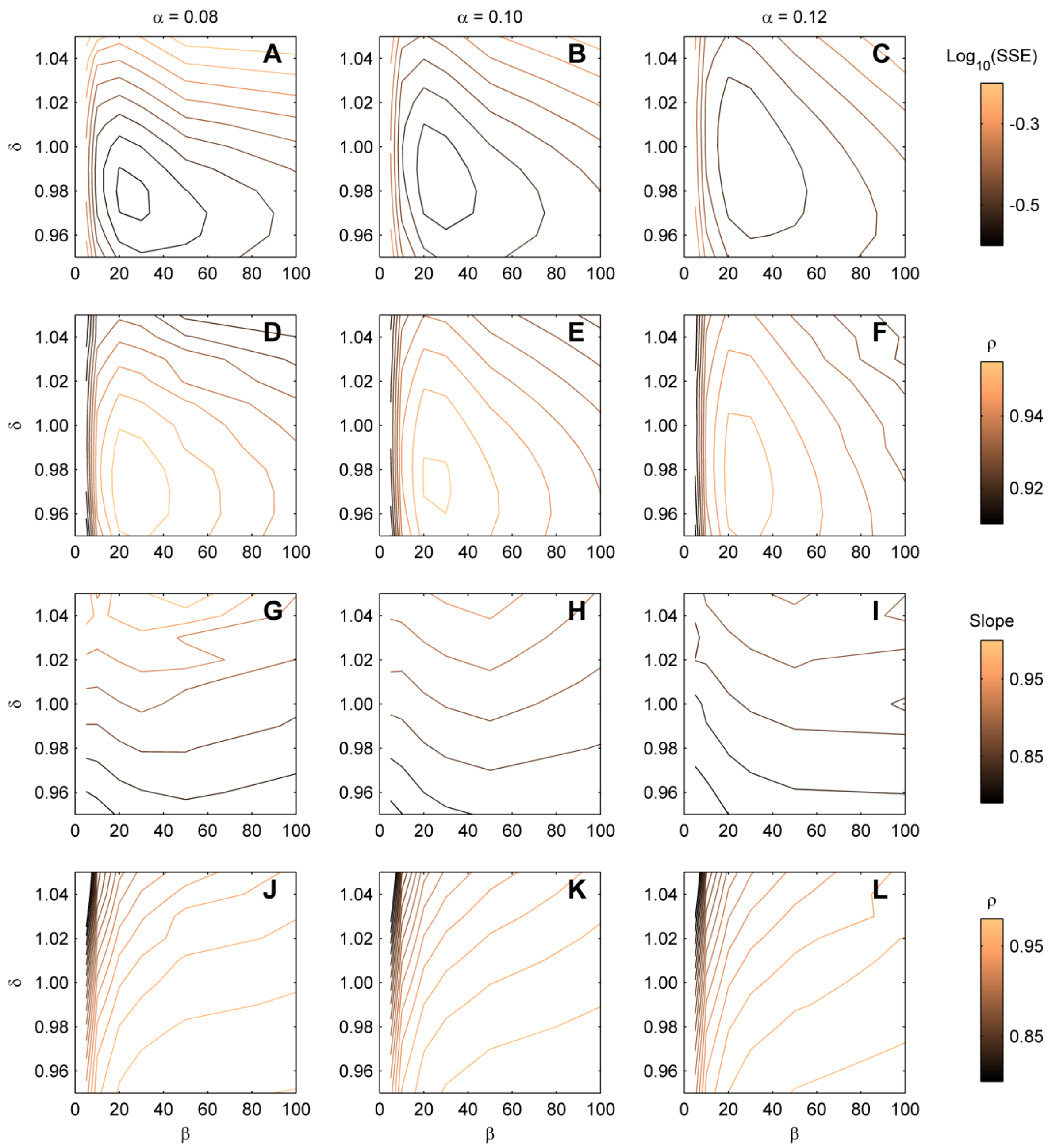

Figure 4. Landscape of different functions of model predictions in the space of the parameters α, β, and δ. (A-C) Sum of squared errors (SSE), (D-F) Pearson's correlation coefficient, and (G-I) slope of the best fit for the amino acid concentration changes. (J-L) Pearson's correlation coefficient between the simulations and experimental measurements for the subset of fluxes used in Moxley et al. [1].

Parameters specific for the analysis of S. cerevisiae's response to WOA treatment

The constructed models for simulating the response of *S. cerevisiae* to treatment with WOAs only have one fitting parameter. This parameter is the uptake rate of the WOA, and it was assumed to be the same for all WOAs. We set the WOA uptake rate that minimizes the average distance of model predictions to the experimental data of the treatment conditions for all WOAs (Figure 5):

$$r_{WOA} = 30.0 r_{WOA,ref}, \quad \text{(S47)}$$

where $r_{WOA,ref}$ is the uptake rate of acetic acid by diffusion in the reference condition, estimated using the method for computing the reference distribution using the procedure described in Section 1 above.

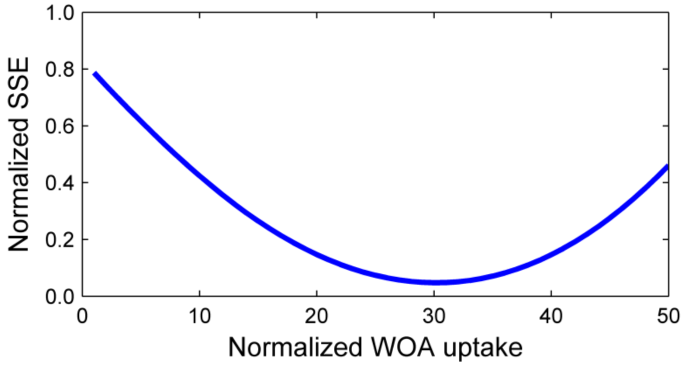

Figure 5. Normalized sum of squared errors (SSE) of model predictions to the experimental data of the treatment conditions as a function of the normalized weak organic acid (WOA) uptake rate. The normalized SSE of the predicted exchange fluxes and biomass yield to the experimental data was normalized with the SSE between the experimental data of the treatment and reference conditions for all WOAs.

Parameters specific for the analysis of S. cerevisiae's response to histidine starvation

The constructed models to simulate the response of *S. cerevisiae* to histidine starvation only have two fitting parameters. The first parameter was included to account for the different inhibition

levels in the wild-type and *gcn4*-knockout cultures because of the different concentrations of 3-aminotriazole used:

$$r_{his} = k_{his}\bar{r}_{his}, \tag{S48}$$

where $\bar{r}_{his}$ denotes the rate expression without accounting for inhibition and the parameter k_{his} was set such that the concentration of histidine matched the experimental measurement.

The second parameter was included to account for the hypothetical inhibition of glycine synthesis from serine by decreasing the concentration of tetrahydrofolate because of the high concentration of 3-aminotriazole used in the wild-type cultures. The formulation of this hypothesis is discussed in the Results Section of the article and computational analysis supporting the hypothesis is given in Section 7 of this Supplementary Materials. Note that the metabolic network did not include the tetrahydrofolate synthesis pathway. Instead, for simplicity, we modeled the inhibition of glycine synthesis from serine by a single parameter:

$$r_{ser\to gly} = k_{ser\to gly}\bar{r}_{ser\to gly}, \tag{S49}$$

where $\bar{r}_{ser\to gly}$ denotes the rate expression without accounting for inhibition and the parameter $k_{ser\to gly}$ was set such that the difference between the predicted and measured concentration changes of glycine and methionine was minimized.

3. Processing of gene expression data

For the analysis of *S. cerevisiae* response under histidine starvation, we used the gene expression data provided as supplemental material in the article by Moxley et al. [1]. The details of the data analysis can be found in the National Center for Biotechnology Information GEO submission GSE 4709. For the analysis of *S. cerevisiae* response to weak organic acids, we used the gene expression provided Abbott et al. [2] in the National Center for Biotechnology Information GEO submission GSE 5926. As Abbott et al., we set the expression values less than 12 to 12 and scaled the microarrays to give an average signal of 150. Then, we smoothed the data using the Lowess method and used the median of three replicates to compute the ratios between treatment conditions and the reference condition.

4. Experimental flux distributions for *Δgcn4* and wild-type cultures

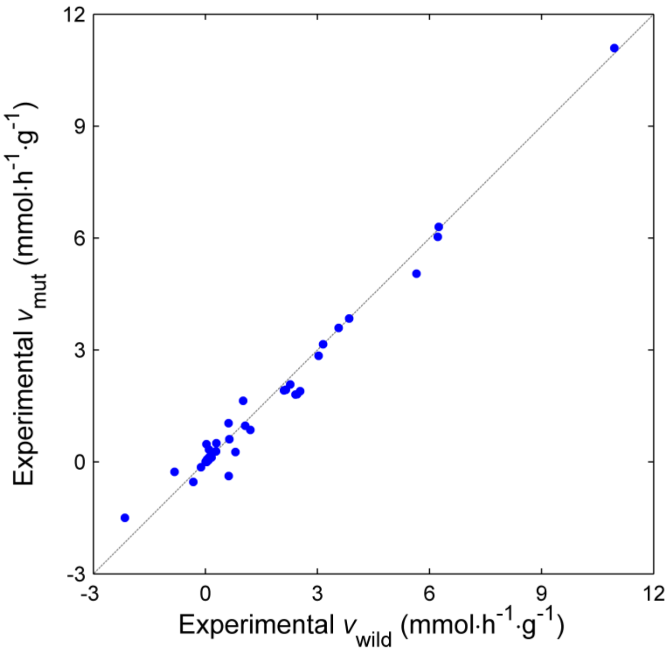

Figure 6. Experimental flux distributions for *Δgcn4* and wild-type cultures as determined by Moxley et al. [1]. The flux distributions were adjusted to satisfy the mass balances of the model used in this work.

5. Simulations under histidine starvation using different values for the constants m_i

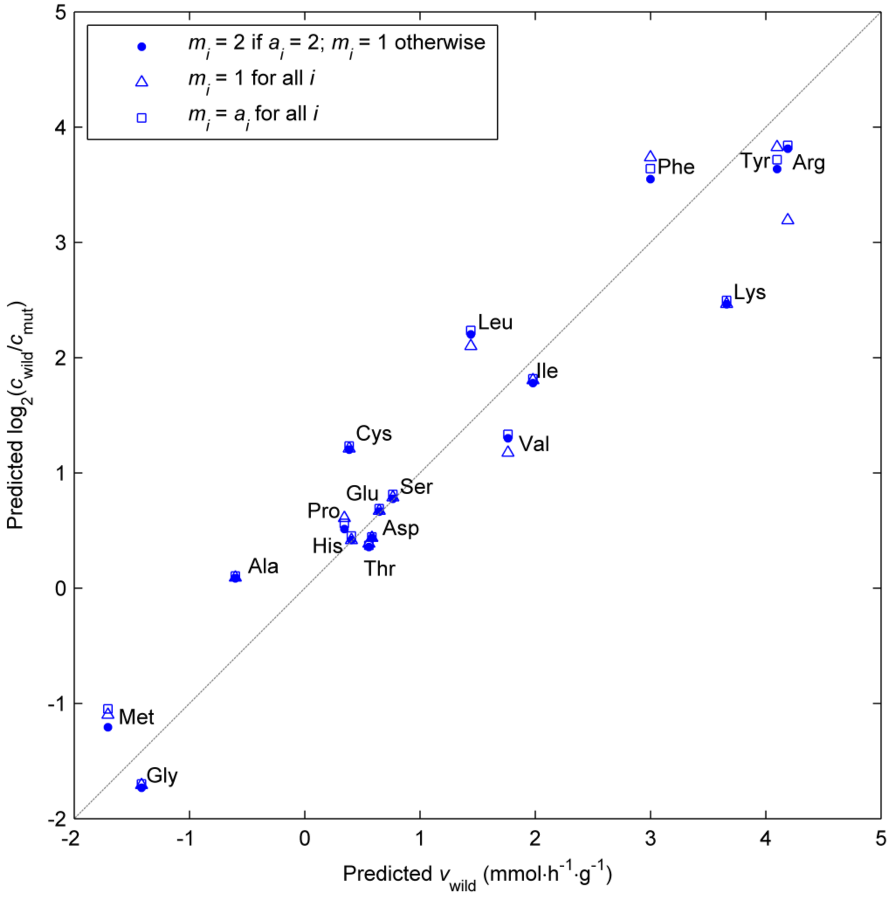

Figure 7. Predicted concentration changes of free amino acids in the analysis of *S. cerevisiae* response to histidine starvation for different choices of the parameters m_i. The choices of the m_i paremeters were $m_i = 2.0$ if $a_i = 2.0$ (dots), $m_i = 1.0$ for all i (triangles), and $m_i = a_i$ for all i (squares). Note that the predicted concentration changes were relatively insensitive to the particular choice of the parameters m_i. The experimental data were taken from Moxley et al. [1].

6. Simulations under histidine starvation without gene expression data

Relatively large correlation coefficients between predicted and experimental free amino acid concentrations can be obtained even without gene expression data. Figure 8 shows the simulated response of the wild-type culture without using gene expression data.

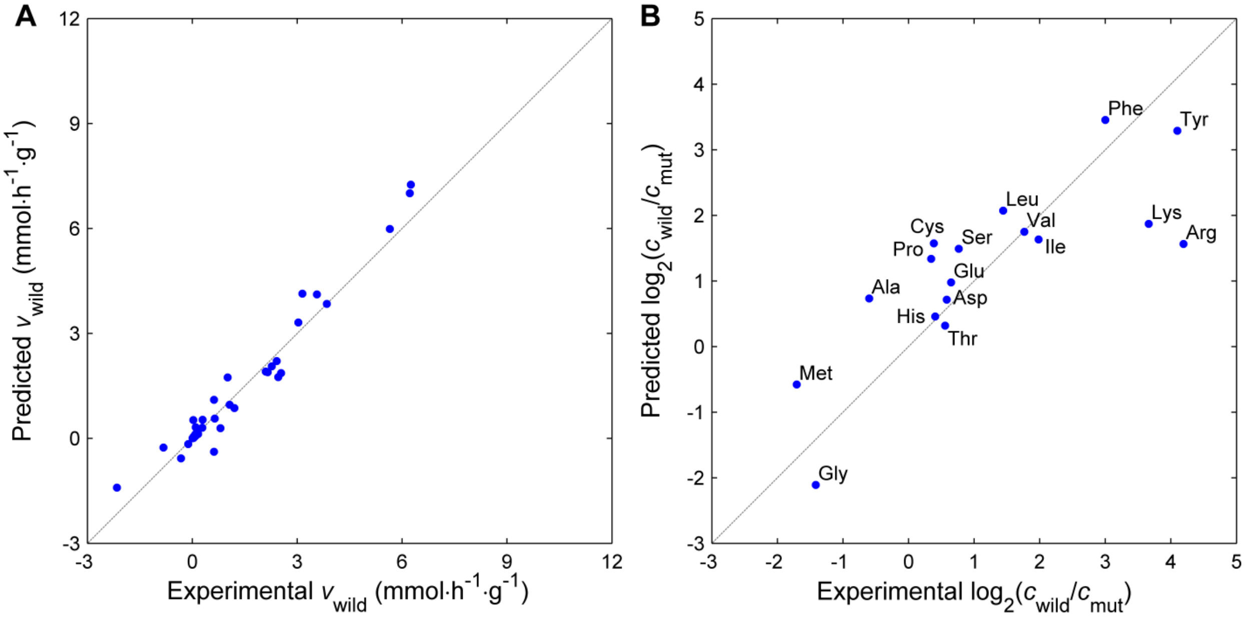

Figure 8. Predicted metabolic response of the wild-type culture treated with 3-aminotriazole without using gene expression data. (**A**) Predicted metabolic fluxes plotted against experimental values. (**B**) Predicted concentration of free amino acids plotted against experimental values. Simulations carried out with the parameters $\alpha = 0.1$, $\beta = 30$, $\delta = 0.97$, $k_{ser \rightarrow gly} = 0.0227$, and $k_{his} = 0.91$. The experimental data were taken from Moxley et al. [1].

7. Identification of candidate off-targets of 3-aminotriazole

We identified candidate off-targets of 3-aminotriazole using an in-house ligand-based target identification protocol [3]. This protocol determines if a query molecule is a potential inhibitor of a protein based on the structural overlap between the query molecule and known inhibitors of the protein. Currently, the database of target proteins contains mainly human proteins. Therefore, we looked for possible targets of 3-aminotriazole in this database. Subsequently, we looked for *S. cerevisiae* proteins with sequence similarity to the identified target human proteins. The top ten proteins with higher score in the database are given in Table 1. Five of the top 10 identified off-target proteins of 3-aminotriazole have high sequence similarity with *S. cerevisiae*'s proteins. Notably, these five proteins are involved in the synthesis of tetrahydrofolate, a coenzyme required for the synthesis of glycine from serine. The genes encoding the potential targets of 3-aminotriazole are shown in the last column of Table 1, and their function in the synthesis of tetrahydrofolate is shown in Figure 9.

Table 1 Identified candidate off-targets proteins of 3-aminotriazole[a]

Target	Species	Off-target score in (%)	*S. cerevisiae* genes
Matrix metalloproteinase-9	*Homo sapiens*	65.35	
Tumor necrosis factor	*Homo sapiens*	65.35	
Dihydrofolate reductase	Several	63.10	YOR236W
Sodium channel protein type 1 subunit alpha	*Homo sapiens*	63.10	
Sodium channel protein type 5 subunit alpha	*Homo sapiens*	63.10	
Hypoxanthine-guanine phosphoribosyltransferase	*Homo sapiens*	62.45	
AMP deaminase 1	*Homo sapiens*	62.45	YML035C
Inosine-5'-monophosphate dehydrogenase 2	*Homo sapiens*	62.45	YHR216W<br>YLR432W<br>YML056C
Inosine-5'-monophosphate dehydrogenase 1	*Homo sapiens*	62.45	YHR216W<br>YLR432W<br>YML056C
Amidophosphoribosyltransferase	*Homo sapiens*	62.45	YMR300C

[a] Shaded rows indicate targets with sequence similarity to *S. cerevisiae*'s proteins, whose encoding genes are shown in the last column.

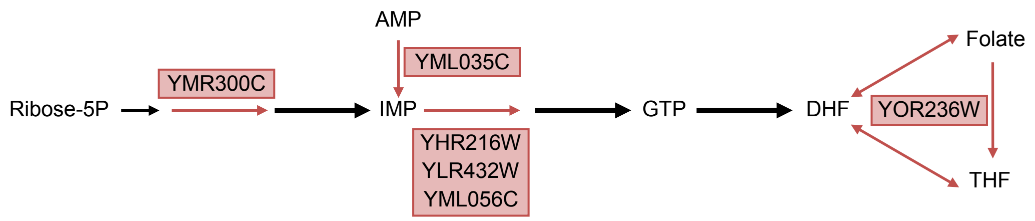

Figure 9. Candidate off-target proteins of 3-aminotriazole in the *S. cerevisiae*. The off-target proteins are indicated by their encoding genes. Thick arrows represent multiple reactions. Abbreviations: ribose-5P, ribose-5-phosphate; AMP, adenosine monophosphate; IMP, inosine monophosphate; GTP, guanosine triphosphate; DHF, dihydrofolate; THF, tetrahydrofolate.

8. Sensitivity analysis of fitting parameters

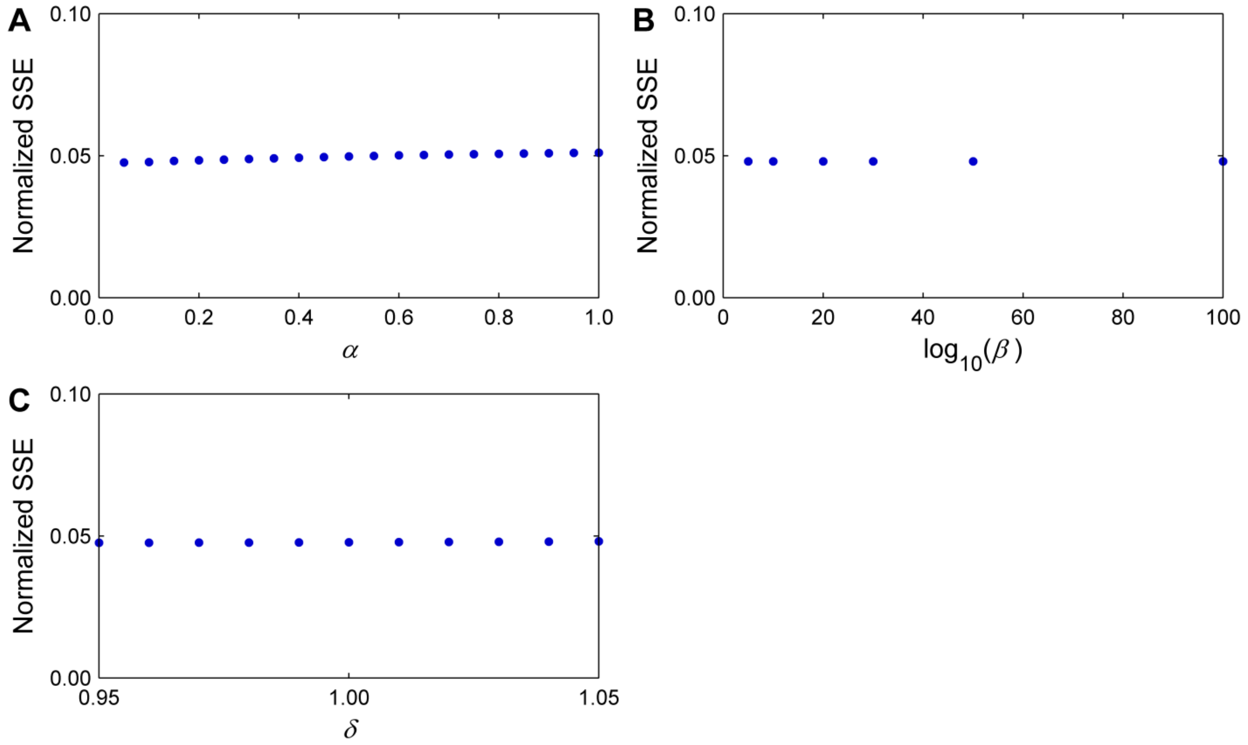

Figure 10. Effect of model parameters on the simulations of the response of *S. cerevisiae* to weak organic acid treatments. SSE denotes the sum of squared errors between predicted exchange fluxes and biomass yield normalized using the SSE between the experimental values for the reference and the corresponding treated culture. (A-C) Normalized SSE as a function of parameters α, β, and δ.

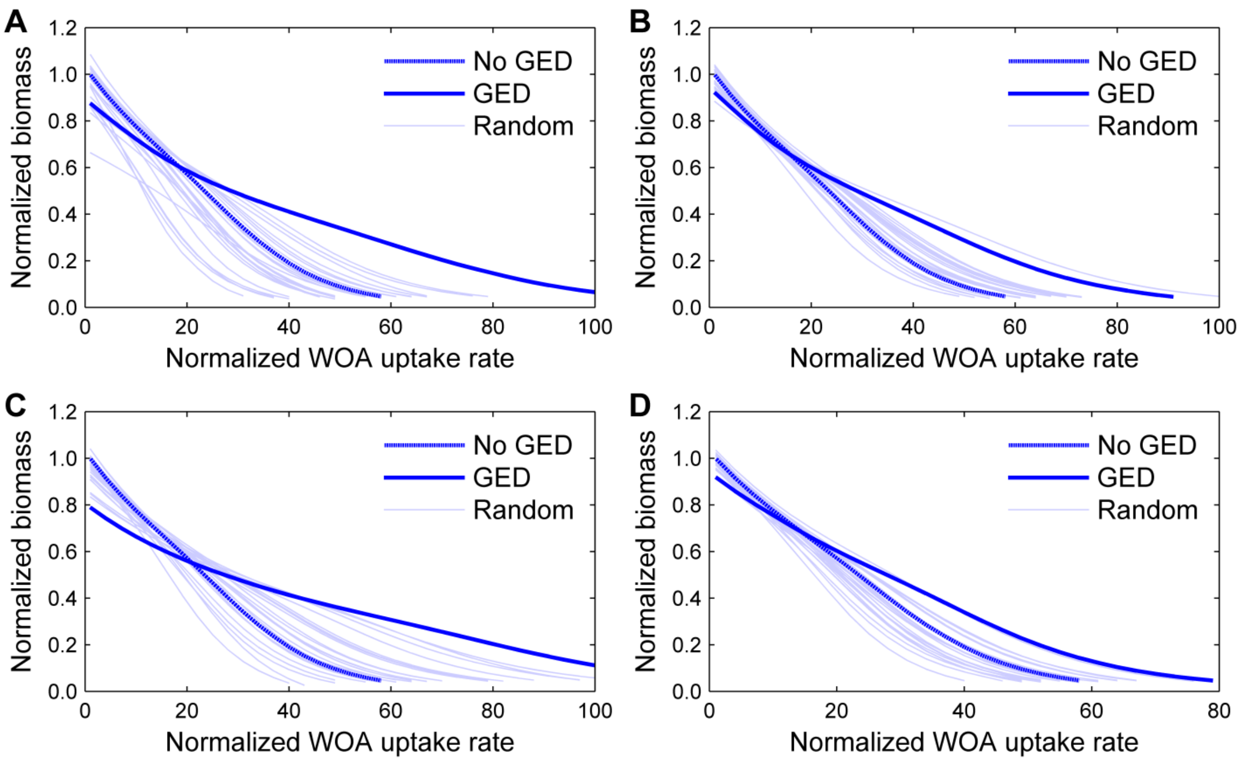

Figure 11. Predicted biomass concentration using randomly generated gene expression data as a function of the WOA uptake rate. The random GED sets were obtained by shuffling the experimental GED. (A) Acetate, (B) benzoate, (C), propionate, and (D) sorbate.

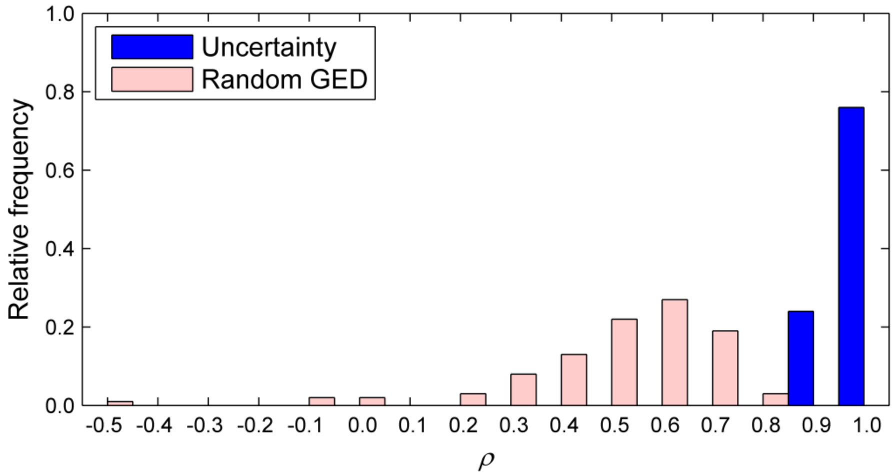

Figure 12. Distribution of the correlation coefficient between predicted and measured concentration changes of free amino acids in the analysis of *S. cerevisiae* response to histidine starvation. The legend "Shuffled" corresponds to simulations carried out by shuffling the GED. The legend "Uncertainty" corresponds to simulation results from uncertainty propagation analysis. The uncertainty propagation analysis was carried out by simulating the model with random metabolic fluxes and GED generated by sampling normal distributions with the mean and standard deviations of the experimental data.

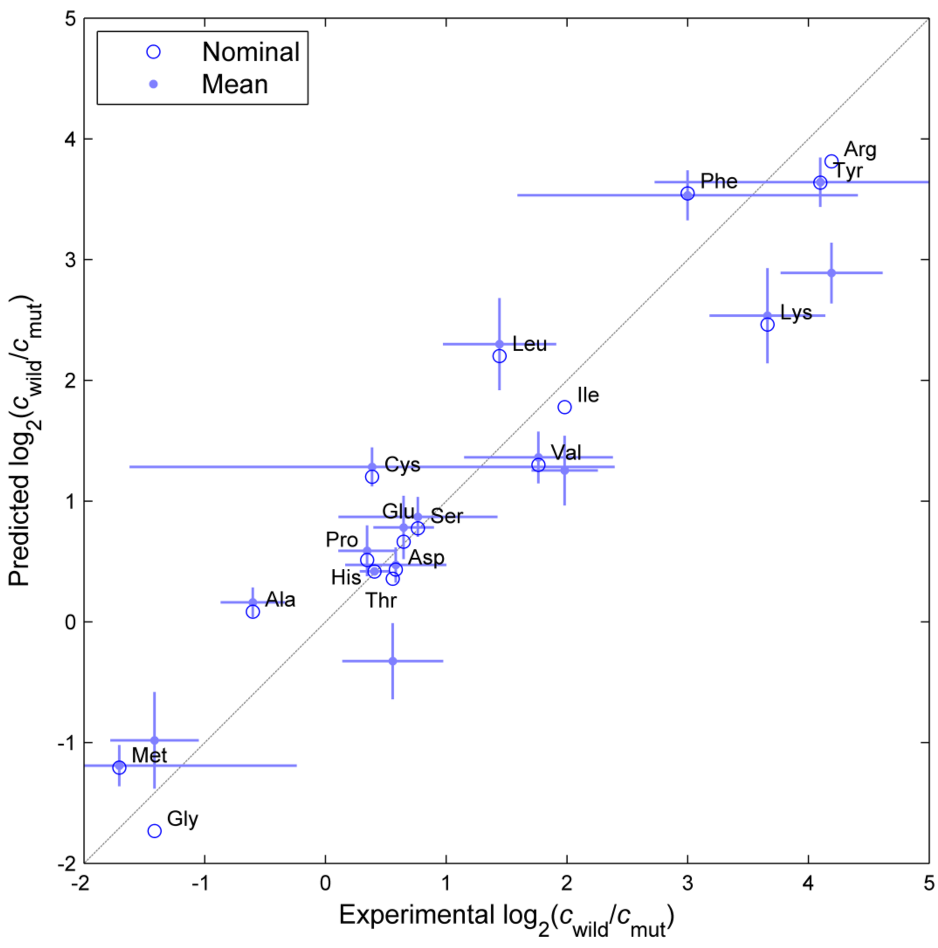

Figure 13. Predicted concentration changes of free amino acids in the analysis of *S. cerevisiae* response to histidine starvation. Bars represent 1.0 standard deviation from the mean concentration changes (dots) for the predictions (vertical bars) and the measurements (horizontal bars). Model predictions using the reported gene expression changes are represented by circles. The uncertainty propagation analysis was carried out by simulating the model with random metabolic fluxes and GED generated by sampling normal distributions with the mean and standard deviations of the experimental data.

9. Expression profiles of the two most determinant reactions for tolerance to weak organic acids (WOAs)

We estimated the gene expression profiles using the gene expression data from Abbott et al. [2]. As in the source article, gene expression levels lower than 12 were set to 12 and all microarrays were scaled to a target average signal of 150. We performed two sample t-test to evaluate differential expression between the treatments and the reference conditions and estimated the false discovery rate on the *p*-values from the t-test. The expression profiles for the genes associated with the two most important reactions of *S. cerevisiae* tolerance to WOA treatment are given in Table 2.

Table 2 Expression profiles of the genes associated with the two most influential reactions for WOA tolerance[a]

Genes	Acetate			Benzoate			Propionate			Sorbate		
	R[b]	*p*[c]	*q*[d]	*R*	*p*	*q*	*R*	*p*	*q*	*R*	*p*	*q*
EC 2.7.1.1 (Glucose → Glucose-6P)												
YDR342C	0.84	0.12	0.19	0.98	0.30	0.23	0.59	0.00	0.03	1.08	0.01	0.04
YDR345C	1.28	0.42	0.34	1.20	0.96	0.44	20.34	0.00	0.00	0.35	0.03	0.06
YHR092C	213.47	0.00	0.05	32.96	0.12	0.14	190.90	0.01	0.05	10.84	0.09	0.11
YHR094C	1.00	0.01	0.07	1.00	0.12	0.14	1.00	0.06	0.09	1.00	0.02	0.05
YHR096C	0.24	0.01	0.08	0.36	0.02	0.07	0.77	0.04	0.07	0.40	0.01	0.04
YMR011W	0.05	0.03	0.10	0.60	0.05	0.10	2.15	0.01	0.04	0.30	0.04	0.07
YCL040W	1.70	0.11	0.18	1.09	0.62	0.34	0.89	0.53	0.29	1.01	0.66	0.33
YFR053C	0.95	0.34	0.31	0.79	0.29	0.23	0.76	0.10	0.11	0.88	0.40	0.24
YGL253W	1.52	0.03	0.11	1.07	0.72	0.37	1.28	0.07	0.10	0.85	0.97	0.41
EC 4.1.1.1 (Pyruvate → Acetaldehyde)												
YLR044C	1.93	0.02	0.09	1.67	0.00	0.05	2.16	0.00	0.03	1.70	0.03	0.07
YGR087C	0.35	0.02	0.10	1.43	0.34	0.24	1.04	0.98	0.42	1.45	0.62	0.32
YLR134W	1.41	0.14	0.20	1.35	0.85	0.41	11.19	0.01	0.04	2.25	0.16	0.14
YDL080C	1.08	0.96	0.53	1.15	0.61	0.34	1.31	0.18	0.15	0.89	0.03	0.07

[a] Shaded cells indicate differentially expressed genes identified following the criteria of Abbott et al. [2] (gene expression change larger than two-fold and a false discovery rate less than 0.5%).
[b] Ratio of gene expression level between the treatment and reference condition.
[c] *p*-values from a two-sample t-test to evaluate differentially expression of genes.
[d] *q*-values for the estimated false discovery rate of differentially expressed genes.

10. References

1. Moxley JF, Jewett MC, Antoniewicz MR, Villas-Boas SG, Alper H, Wheeler RT, Tong L, Hinnebusch AG, Ideker T, Nielsen J, Stephanopoulos G: **Linking high-resolution metabolic flux phenotypes and transcriptional regulation in yeast modulated by the global regulator Gcn4p.** *Proc Natl Acad Sci U S A* 2009, **106:**6477-6482.
2. Abbott DA, Knijnenburg TA, de Poorter LM, Reinders MJ, Pronk JT, van Maris AJ: **Generic and specific transcriptional responses to different weak organic acids in anaerobic chemostat cultures of *Saccharomyces cerevisiae*.** *FEMS Yeast Res* 2007, **7:**819-833.
3. AbdulHameed MD, Chaudhury S, Singh N, Sun H, Wallqvist A, Tawa GJ: **Exploring polypharmacology using a ROCS-based target fishing approach.** *J Chem Inf Model* 2012, **52:**492-505.
